# Secure and scalable deduplication of horizontally partitioned health data for privacy-preserving distributed statistical computation

**DOI:** 10.1186/s12911-016-0389-x

**Published:** 2017-01-03

**Authors:** Kassaye Yitbarek Yigzaw, Antonis Michalas, Johan Gustav Bellika

**Affiliations:** 1 Department of Computer Science, UiT The Arctic University of Norway, 9037 Tromsø, Norway; 2 Norwegian Centre for E-health Research, University Hospital of North Norway, 9019 Tromsø, Norway; 3 Department of Computer Science, University of Westminster, 115 New Cavendish Street, London, W1W 6UW UK; 4 Department of Clinical Medicine, UiT The Arctic University of Norway, 9037 Tromsø, Norway

**Keywords:** Bloom Filter, Data Reuse, Deduplication, Distributed Statistical Computation, Data Linkage, Duplicate Record, Electronic Health Record, Privacy, Record Linkage, Set Intersection

## Abstract

**Background:**

Techniques have been developed to compute statistics on distributed datasets without revealing private information except the statistical results. However, duplicate records in a distributed dataset may lead to incorrect statistical results. Therefore, to increase the accuracy of the statistical analysis of a distributed dataset, secure deduplication is an important preprocessing step.

**Methods:**

We designed a secure protocol for the deduplication of horizontally partitioned datasets with deterministic record linkage algorithms. We provided a formal security analysis of the protocol in the presence of semi-honest adversaries. The protocol was implemented and deployed across three microbiology laboratories located in Norway, and we ran experiments on the datasets in which the number of records for each laboratory varied. Experiments were also performed on simulated microbiology datasets and data custodians connected through a local area network.

**Results:**

The security analysis demonstrated that the protocol protects the privacy of individuals and data custodians under a semi-honest adversarial model. More precisely, the protocol remains secure with the collusion of up to *N* − 2 corrupt data custodians. The total runtime for the protocol scales linearly with the addition of data custodians and records. One million simulated records distributed across 20 data custodians were deduplicated within 45 s. The experimental results showed that the protocol is more efficient and scalable than previous protocols for the same problem.

**Conclusions:**

The proposed deduplication protocol is efficient and scalable for practical uses while protecting the privacy of patients and data custodians.

**Electronic supplementary material:**

The online version of this article (doi:10.1186/s12911-016-0389-x) contains supplementary material, which is available to authorized users.

## Background

Electronic health record (EHR) systems have been in existence for many years. The increased adoption of EHR systems has led, and continues to lead, to the collection of large amounts of health data [1]. Large amounts of administrative, survey, and registry data are also being collected. These data could aid in the development of scientific evidence that helps improve the effectiveness, efficiency, and quality of care of healthcare systems [2–4].

### Introduction

The focus of this paper is the reuse of health data horizontally partitioned between data custodians, such that each data custodian provides the same attributes for a set of patients. Reusing data from multiple data custodians provides a sufficient number of patients who satisfy the inclusion criteria of a particular study. The number of patients at a single data custodian may provide insufficient statistical power, especially for studies on rare exposures or outcomes. When data are collected across multiple data custodians, the data of a heterogeneous mix of patients can be reused.

There has been substantial interest in the reuse of EHR data for public health surveillance, which also requires data from multiple data custodians covering the geographic area of interest [5–7]. One of the EHR meaningful use criteria in the United States is the ability to release health data for public health surveillance [8].

**Fig. 1:**
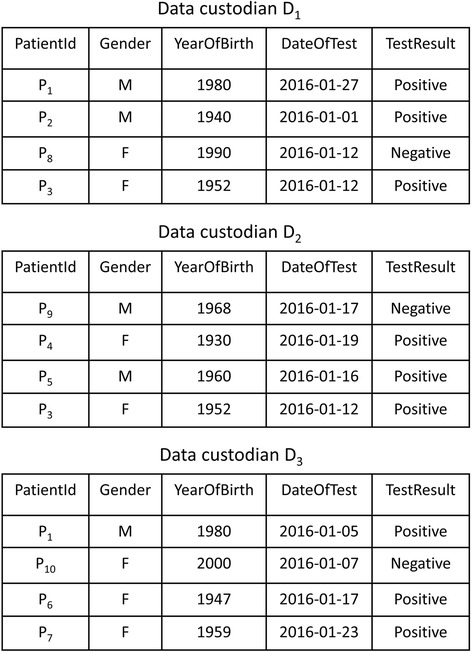
A simplified virtual dataset of influenza A test results distributed across three data custodians

The horizontally partitioned datasets required for a health study or disease surveillance are often queried by distributing the query to data custodians, who execute the query and store a copy of the data extracts locally [9]. We refer to the data extracts distributed across data custodians as a virtual dataset (VD). Consider the execution of the query “select the records of patients tested for influenza A viruses in January 2016” across three data custodians $\mathcal{D}=\left\{{D}_1,{D}_2,{D}_3\right\}$. Figure 1 illustrates a VD that consists of the query results for the data custodians.

A VD may contain duplicate records from data custodians that cover overlapping areas and areas in close proximity [10–12]. The duplicate records can be exact or approximate. A set of records are exact duplicates if the records are compared using exact comparison functions, and the records have the same value for all attributes used for comparison. In contrast, approximate duplicate records are compared using comparison functions that allow approximate similarities, and the records have different values for one or more attributes.

#### Privacy-preserving distributed statistical computation

Access to and the use of patient data for research raise significant privacy concerns for the various stakeholders (i.e., patients and data custodians) [7, 13, 14]. A recent approach that addresses privacy concerns is secure multi-party computation (SMC), which deals with the problem of computing a function *f* on distributed data without revealing any information except the results. SMC protocols have been developed for the statistical computation of distributed data that do not reveal anything except the results [15–21].

Statistical analysis of a virtual dataset that contains duplicate records may lead to incorrect results. Let us consider a query of the number of patients in a VD that satisfy a set of criteria. When there are duplicate records in the VD, a simple summation of the data custodians’ local counts will not return the same result if the query is run against the combined datasets of all data custodians stored in a central database.

For example, the distributed statistical computation of the number of women who tested positive for influenza A against the VD shown in Fig. 1 would return an incorrect result. Patient P_3_ would be counted twice, as she has a positive test result stored in *D*_1_ and *D*_2_. Therefore, to improve the accuracy of the statistical results, deduplication of the VD is a necessary preprocessing step before statistical analysis is conducted.

#### Privacy-preserving deduplication

Deduplication (also known as record linkage) is the process of linking records at the same or different data custodians that refer to the same individual. In contrast to record linkage, the final goal of deduplication is to remove duplicate records while maintaining a single occurrence of each record. Privacy-preserving record linkage (PPRL; also known as private set intersection and private record linkage) protocols have been developed to link records across multiple data custodians without revealing any information other than the linkage result [22, 23]. The main challenges of these protocols for practical use include the quality of the linkage, privacy, efficiency, and scalability [22].

The objective of this paper is to develop an efficient and scalable protocol for the deduplication of a VD while protecting the privacy of the patients and the data custodians. The proposed protocol supports various deterministic record linkage algorithms.

Our main contributions can be summarized as follows: We propose a novel efficient and scalable protocol based on Bloom filters for the privacy-preserving deduplication of a horizontally partitioned dataset. We provide proof of the security of the protocol against a semi-honest adversarial model in which the participating entities are assumed to follow the protocol steps, but the entities may try to learn private information from the messages exchanged during the protocol execution. We conducted a theoretical analysis of the protocol’s efficiency and scalability. We implemented a prototype of the protocol and ran experiments among three microbiology laboratories located in Norway. We also ran experiments using simulated microbiology laboratory datasets with up to 20 data custodians and one million records.

The remainder of this section presents a review of related work and provides a use case for the deduplication problem and formally presents it. The Methods section outlines the requirements of the proposed protocol, as well as the threat model and assumptions, Bloom filter, notations, basic set operations, and secure sum protocol used in the protocol. Then, the proposed protocol is described. The Results section presents the security analysis, implementation, and evaluations of the protocol. Finally, the Discussion and Conclusions are presented.

### Related work

Several PPRL protocols have been developed based on either deterministic or probabilistic matching of a set of identifiers. Interested readers are referred to [22, 23] for an extensive review of the PPRL protocols. The protocols can be broadly classified as protocols with or without a third party. In this section, we review privacy-preserving protocols for deterministic record linkage. These protocols are secure against the semi-honest adversarial model, which is the adversarial model considered in this paper.

A record contains a set of identifiers that consists of direct and indirect (quasi-identifier) identifiers and other health information. Direct identifiers are attributes that can uniquely identify an individual across data custodians, such as a national identification number (ID). In contrast, quasi-identifiers are attributes that in combination with other attributes can identify an individual, such as name, sex, date of birth, and address. In this paper, the terms identifier and quasi-identifier are used interchangeably.

Weber [12] and Quantin et al. [24] proposed protocols that use keyed hash functions. These protocols require data custodians send a hash of their records’ identifiers to a third party that performs exact matching and returns the results. The data custodians use a keyed hash function with a common secret key to prevent dictionary attacks by the third party. These protocols are secure as long as the third party does not collude with a data custodian. Quantin et al.’s protocol [24] performs phonetic encoding of the identifiers (i.e., last name, first name, date of birth, and sex) before hashing, in order to reduce the impact of typing errors in the identifiers on the quality of the linkage.

Several protocols were proposed based on commutative encryption schemes^1^ [25–27]. In these protocols, each data custodian, in turn, encrypts the unique identifiers for all records across the data custodians using its private key, and consequently, each unique identifier is encrypted with the private keys of all the data custodians. Then, the encrypted unique identifiers are compared with each other, as the encrypted values of two unique identifiers match if the two unique identifiers match. The protocols proposed in [25, 26] are two-party computation protocols, whereas Adam et al.’s [27] protocol is a multi-party computation protocol.

The protocols reviewed thus far require the exchange of a long list of hash or encrypted identifiers, which can limit the scalability of the protocols as the number of data custodians and records increases. In addition, protocols based on commutative encryption require communication rounds quadratic with the number of data custodians.

Multi-party private set intersection protocols were designed based on Bloom filters^2^ [28, 29]. In general, each data custodian encodes the unique identifier values of its records as a Bloom filter (see the description of a Bloom filter in the Methods section). The protocols use different privacy-preserving techniques, as discussed below, to intersect the Bloom filters and then create a Bloom filter that encodes the unique identifiers of the records that have exact matches at all data custodians. Then, the data custodian queries the unique identifiers of its records in the intersection Bloom filter to identify the records that match.

In Lai et al.’s [28] protocol, each data custodian splits its Bloom filter into multiple segments and distributes them to the other participating data custodians while keeping one segment for itself. Then, each data custodian locally intersects its share of the Bloom filter segments and distributes it to the other data custodians. Finally, the data custodians combine the results of the intersection of the Bloom filter segments to create a Bloom filter that is an intersection between all the data custodians’ Bloom filters. The protocol requires communication rounds quadratic with the number of data custodians, and the protocol is susceptible to a dictionary attack of the unique identifiers that have all the array positions in the same segment of the Bloom filter.

In Many et al.’s [29] protocol, each data custodian uses secret sharing schemes^3^ [30] to split each counter position of the data custodian’s Bloom filter and then distributes them to three semi-trusted third parties. The third parties use secure multiplication and comparison protocols to intersect the data custodians’ Bloom filters, which adds overhead to the protocol.

Dong et al. [31] proposed a two-party protocol for private set intersection. The protocol introduced a new variant of a Bloom filter, called a garbled Bloom filter, using a secret sharing scheme. The first data custodian encodes the unique identifiers of the data custodian’s records as a Bloom filter, whereas the second data custodian encodes the unique identifiers of its records as a garbled Bloom filter. Then, the data custodians intersect their Bloom filters using an oblivious transfer technique (OT)^4^ [32], which adds significant overhead to the overall performance of the protocol.

Karapiperis et al. [33] proposed multi-party protocols for a secure intersection based on the Count-Min sketch.^5^ Each data custodian locally encodes the unique identifiers of its records based on the Count-Min sketch, denoted as the local synopsis, and then, the data custodians jointly compute the intersections of the local synopses using a secure sum protocol. The authors proposed two protocols that use secure sum protocols based on additive homomorphic encryption [34] and obscure the secret value with a random number [19, 35]. The protocols protect only the data custodians’ privacy, whereas our protocol protects individuals’ and data custodians’ privacy. The additive homomorphic encryption adds computation and communication overhead as the number of records and data custodians increases.

The results of the protocols in [28, 29, 31, 33] contain the probability of a false positive. Although the protocols can choose a small false positive probability, for some applications, a false positive probability may not be acceptable.

### Use case

The need for comprehensive and timely infectious disease surveillance is fundamental for public health monitoring that makes early prevention and control of disease outbreaks possible. EHRs have been used as a data source for routine syndromic and laboratory-based public health surveillance [5–7].

The use case considered in this paper is distributed disease surveillance [6]. In particular, we consider the Snow system that is used for experimental evaluations of the protocol proposed in this paper [36]. The Snow system uses microbiology laboratory test results from multiple microbiology laboratories in Norway. The laboratories collect and analyze samples from patients in primary care settings, such as general practitioner offices and nursing homes.

Every day, the Snow system extracts anonymized test results and maintains the datasets within local databases at each laboratory according to a predefined data model. The data extracts contain attributes, such as infectious agent, age, sex, geographic area, and time. The Snow system broadcasts a query across the laboratories and reveals only the number of matching patients at each laboratory. We extend the Snow system with a secure sum protocol to hide the local count of a single laboratory [20].

Consider the statistical query of the count of positive or negative test results for a disease in a particular stratum of individuals (e.g., male or female) within a VD. A simple summation of the laboratories’ local counts gives an overestimated count when the test results are duplicated across the laboratories. A laboratory may transfer test samples to another laboratory when the first laboratory does not have the appropriate laboratory equipment. Then, when the test results are sent to the first laboratory, the same test result appears in both laboratories’ datasets.

In the context of infectious disease surveillance, two or more separate tests for an individual that have a positive result can also be considered duplicates depending on the required aggregate query for the dataset, such as the number of patients who have had a particular disease and the number of disease episodes.

Individuals may be infected with the same disease multiple times within a given time period, which may lead to being tested for the same disease at multiple laboratories. Individuals can also be tested at multiple laboratories for the same infection; this practice is more common in chronic infections. Testing at multiple laboratories may occur when patients switch healthcare providers, receive emergency care, or visit different providers during an episode of infection [37]. In Norway, primary care institutions may send samples collected from a patient to different laboratories, and patients can change general practitioners up to twice a year.

Consider a statistical query of the number of individuals infected with influenza A viruses within the VD shown in Fig. 1. The query requires that patient P_1_ is counted once, even if the patient has two positive test results at data custodians *D*_1_ and *D*_3_. For this query, the objective of the deduplication is to link the positive test results for each individual in the VD and to maintain the test result at only one of the laboratories.

When the number of disease episodes is counted, the number of positive test results for different disease episodes for an individual across the laboratories is counted separately. However, the positive test results for an individual in the same disease episode should be counted once. For example, Lazarus et al. [38] grouped two healthcare service encounters for a patient for a lower respiratory infection into one episode if the subsequent visit occurred within six weeks of the preceding visit. The researchers assumed that the second visit likely represented a follow-up visit for the same infection. In this context, the objective of deduplication is to link an individual’s positive test results for the same disease episode and keep the test result at only one of the laboratories.

We describe the protocol proposed in this paper in the context of deduplicating a VD to be able to accurately compute the statistical count of the number of individuals infected with the disease in question. However, the protocol can be easily extended to other types of statistical count queries.

### Problem statement and definitions

In this section, we define the context for the deduplication problem and the problem statement.

#### Data custodian (*D*_*i*_)

We assume three or more data custodians (e.g., hospitals, general practitioner offices, or medical laboratories) are willing to share their data for a secondary use in a health study but are concerned about privacy risks. The data custodians form a distributed health research network denoted by $\mathcal{D}=\left\{{D}_1,{D}_2,\dots, {D}_N\right\}$, where *D*_*i*_ is a data custodian.

#### Data schema

The heterogeneity of data models is a challenge in reusing data from multiple data custodians [39]. Therefore, the distributed data must be harmonized through standardization. For example, several distributed health research networks, such as Mini-Sentinel [40] and the Shared Health Research Information Network (SHRINE) [41], create a common data model by transforming the data at each data custodian into a predefined common data model and data representations [9].

In this paper, for simplicity, we assume a common data model exists across the data custodians that enforces uniform attribute naming conventions, definitions, and data storage formats. We also assume the data distributed across the data custodians are horizontally partitioned in that each data custodian *D*_*i*_ collects the same attributes for a set of patients.

#### Virtual dataset (VD)

We assume the data query for a particular study can be broadcast to all data custodians $\mathcal{D}$. Then, each data custodian executes the query and stores a copy of the query result locally. The data extracts across the data custodians form a virtual dataset. We make the same assumption as above that the VD adheres to a common data model.

#### Record linkage

We consider deterministic record linkage algorithms in which a set of records belongs to the same person if they exactly or partially match on a predefined combination of identifiers. First, we describe the protocol proposed in this paper by assuming the existence of a common unique identifier *j* for a patient denoted by *p*_*j*_. Second, we extend the protocol for deterministic record linkage using quasi-identifiers, when the available unique identifier is low quality or does not exist.

#### Problem statement

Assume a subset of data custodians ${\mathcal{D}}_s\subseteq \mathcal{D}$. Each data custodian ${D}_i\in {\mathcal{D}}_s$ has a record *r*_*j*_ of patient *p*_*j*_ in a virtual dataset. The problem addressed in this paper is to find a privacy-preserving protocol through which the patient’s duplicate records are identified and removed from the virtual dataset while one occurrence of the record is maintained at one of the data custodians.

## Methods

### Overview

**Fig. 2:**
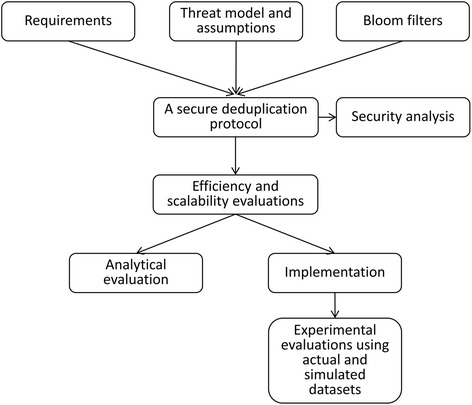
An overview of the methods for developing and evaluating the proposed protocol

Figure 2 shows an overview of the methods we used to develop and evaluate the secure deduplication protocol proposed in this paper. First, we defined the requirements for the protocol and the threat model and assumptions with which the protocol would be secure. We presented the building blocks used in the protocol, such as a Bloom filter, functions for the basic operations of Bloom filters, and secure sum protocol, and described the proposed protocol.

We then performed a security analysis of the proposed protocol. We also conducted theoretical and experimental evaluations of the protocol’s efficiency and scalability. We implemented a prototype of the protocol and ran the experiments on the virtual datasets distributed across three Norwegian microbiology laboratories. We also ran experiments on simulated datasets with up to 20 data custodians and one million records.

### Requirements for secure deduplication protocol

Data custodians’ privacy concerns about disclosing patient data continue, even in the context of a pandemic [42]. Therefore, a deduplication protocol should protect the privacy of patients who have records in a VD.

However, even when patients’ privacy is protected, data custodians (e.g., clinicians and health institutions) have expressed concerns about their own privacy risks [7]. For example, deduplication may reveal the total number of patients in a data custodian who satisfy certain criteria. Although this information does not directly reveal any information about the patients, data custodians might consider this information sensitive, and in many scenarios, it needs to be hidden.

For example, a general practitioner may fear that the number of laboratory test requests and results she sent to laboratories could be used to evaluate her testing behavior. A microbiology laboratory may fear that other laboratories and investors may use the number of tests the laboratory performs during a time period to gain competitive advantage. Therefore, the protocol should be designed in such a way that the total number of patients remains private.

The protocol should allow only a data custodian to learn which of its records have duplicates in the VD, which does not reveal any new sensitive information to the data custodian. However, the identity of the data custodians that contributed the duplicate records should remain unknown.

For example, in Fig. 1, the influenza A–positive test results for patient P_1_ are stored at *D*_1_ and *D*_3_. Data custodian *D*_2_ cannot learn any information about P_1_. *D*_1_ and *D*_3_ learn only that P_1_ tested positive for influenza A at another anonymous laboratory, which is not sensitive information.

Often, public health studies require a large number of patients’ data from several data custodians. Therefore, the deduplication protocol should be computationally efficient and scale with the number of records and participating data custodians.

### Threat model and assumptions

We considered a semi-honest (honest-but-curious) adversarial model in which the data custodians correctly follow the protocol specifications using the data custodians’ correct data. However, the data custodians might use the messages exchanged during the protocol execution to learn information that otherwise should remain private. The adversarial model allows efficient and scalable protocols, whereas the malicious adversarial model provides stronger security at the expense of significant computation and communication costs [43–46].

We also assumed that a semi-trusted third party (STTP), denoted as the *coordinator*, who participates in the protocol without any input. In addition, we assumed that the *coordinator* follows the protocol specification and does not collude with a data custodian. An efficient and scalable protocol can be constructed using an STTP [7, 47].

We assumed that the communications between two entities that participate in the protocol are secure. Therefore, an adversary cannot read the messages sent between two honest entities, and the integrity of the messages is verified.

### Bloom filter

A Bloom filter (*BF*) is a space-efficient probabilistic data structure that encodes a set $\mathcal{X}$ of *n* elements [48]. A *BF* is an array of size *m*, and each array position has one bit initially set to 0. The Bloom filter allows insertion and membership queries of an element $x\in \mathcal{X}$.

Bloom filter operations are performed using *k* independent hash functions *H*_*h*_(.), where 1 ≤ *h* ≤ *k*. First, the hash of an element *x* is computed using each hash function *H*_*h*_(*x*). Second, the modulo *m* of each hash value is computed to get *k* array positions of *BF*, *b*_*h*_(*x*) = *H*_*h*_(*x*) *mod m*, where *b*_*h*_(*x*) ∈ [0, *m* − 1]. Then, *x* is inserted into the *BF* by setting all the positions *b*_*h*_(*x*) of *BF* to 1. The element *x* is concluded to be a non-member of the *BF* if at least one of the positions *b*_*h*_(*x*) of the *BF* is 0.

**Fig. 3:**
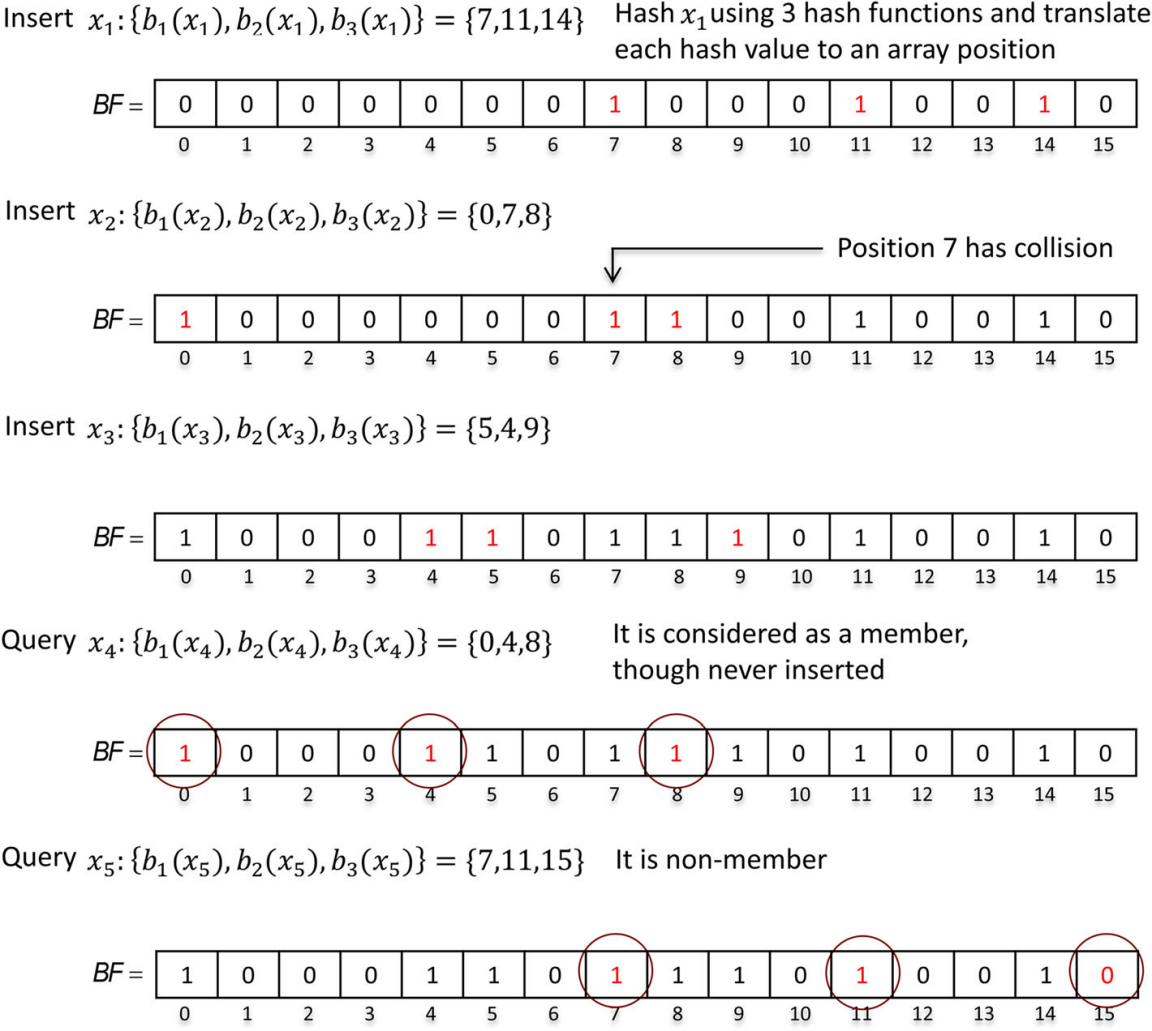
Insertion and membership query for a Bloom filter (BF) (*m* = 16, *k* = 3). *b*_*i*_(*x*_*j*_) denotes an array position for *x*_*j*_ with a hash function denoted as *H*_*i*_(.)

A membership query result can be a false positive due to the hash collisions that occur when all the positions *b*_*h*_(*x*) of the *BF* have been set to 1 as a result of the insertion of other elements. After elements equal to the expected number of elements *n* are inserted into the *BF* the false positive probability of a membership query is equal to *P*(*false positve*) ≈ (1 − *e* ^− *kn*/*m*^)^*k*^ [49]. Figure 3 presents an example of a Bloom filter through inserting and querying elements.

A counting Bloom filter (*CBF*) is an extension of a Bloom filter [49, 50] that supports the deletion of elements, as well as insertion and membership queries. Each array position of a CBF has a counter size *c* greater than one bit that is large enough to avoid counter overflow.

An element *x* is inserted into the *CBF* by incrementing all the counters at the array positions *b*_*h*_(*x*) of the *CBF* by 1. Similarly, an element *x* is deleted from the *CBF* by decrementing all the counters at positions *b*_*h*_(*x*) by 1. The element *x* is concluded to be a non-member of the *CBF* if at least one of the positions *b*_*h*_(*x*) is 0. A membership query has the same false positive probability as a Bloom filter.

### Notations

In this section, we describe the notations that are used in the remainder of the paper. *I*_*i*_ denotes a set of the unique identifiers (IDs) of the records of a data custodian ${D}_i\in \mathcal{D}$ in a particular deduplication query. The union of all the IDs of the records across all data custodians is denoted as *S* = *I*_1_ ∪ *I*_2_ ∪ … ∪ *I*_*N*_. We write *S* ∩ *I*_*i*_ and *S* ∪ *I*_*i*_ to denote the intersection and the union between sets *S* and *I*_*i*_, respectively.

We use *CBF*_*s*_ to denote a counting Bloom filter that encodes set *S* and use *CBF*_*I*_^*i*^ and *BF*_*I*_^*i*^ to denote a counting Bloom filter and a Bloom filter that encode set *I*_*i*_, respectively. *CBF*_*S* ∩ *I*_^*i*^ and *CBF*_*S* ∪ *I*_^*i*^ encode sets *S* ∩ *I*_*i*_ and *S* ∪ *I*_*i*_, respectively.

We use *CBF*_*r*_^*i*^ to denote the random counting Bloom filter of data custodian *D*_*i*_ and use *CBF*_*R*_ to denote the sum of all the random counting Bloom filters, ${\displaystyle {\sum}_{i=1}^NCB{F}_r^i}$. However, *CBF*_*R*_^*i*^ denotes the partial sum of the random counting Bloom filters, ${\displaystyle {\sum}_{j=1}^iCB{F}_r^j}+CB{F}_r^0$, where *CBF*_*r*_^0^ denotes the initial random counting Bloom filter of the leader data custodian *D*_*L*_.

The union of *CBF*_*I*_^*i*^ and *CBF*_*r*_^*i*^ is denoted as *CBF*_*r* ∪ *I*_^*i*^, and the Bloom filter representation of *CBF*_*r* ∪ *I*_^*i*^ is denoted as *BF*_*r* ∪ *I*_^*i*^. *CBF*_*R* ∪ *S*_ denotes the union of *CBF*_*R*_ and *CBF*_*S*_.

### Set operations on Bloom filters

Table 1 describes the main functions for the set operations on Bloom filters that are required for the construction of our protocol. Interested readers are referred to Additional file 1 for detailed descriptions and the algorithms of the functions.

**Table 1:** Functions for basic operations of Bloom filters

Functions	Description
add(*CBF*_*r*_^*i*^, *CBF*_*I*_^*i*^)	Returns counting Bloom filter *CBF*_*r* ∪ *I*_^*i*^ that represents the summation of *CBF*_*r*_^*i*^ and *CBF*_*I*_^*i*^
sub(*CBF*_*R* ∪ *S*_, *CBF*_*R*_)	Returns counting Bloom filter *CBF*_*S*_ that represents the subtraction of *CBF*_*R*_ from *CBF*_*R* ∪ *S*_
intersect(*CBF*_*S*_, *BF*_*I*_^*i*^)	Returns counting Bloom filter *CBF*_*S* ∩ *I*_^*i*^ that represents the intersection between *CBF*_*S*_ and *BF*_*I*_^*i*^
count(*CBF*_*S* ∩ *I*_^*i*^, {*b*_1_(*x*), *b*_2_(*x*), …, *b*_*k*_(*x*)})	Returns *f* that is equal to the number of occurrences of *x* in *CBF*_*S* ∩ *I*_^*i*^
toBloomFilter(*CBF*_*I*_^*i*^)	Returns Bloom filter *BF*_*I*_^*i*^ that represents *CBF*_*I*_^*i*^

### Secure sum protocol

Several secure sum protocols are constructed using different building blocks [7, 17, 51, 52]. Secure sum protocols compute $s={\displaystyle {\sum}_{i=1}^N{v}_i}$, where *v*_*i*_ ∈ [0, *m*) is the secret value of data custodian *D*_*i*_. The protocols compute without disclosing *v*_*i*_ to any entity that participates in the protocol. We extend the secure sum protocol proposed in [19, 35] to compute the union of random counting Bloom filters, $CB{F}_R={\displaystyle {\sum}_{i=1}^NCB{F}_r^i}$, where *CBF*_*r*_^*i*^ is the random counting Bloom filter of *D*_*i*_. Assume that *D*_1_ is selected as the leader data custodian, denoted as *D*_*L*_. The protocol steps are shown in Algorithm 1.
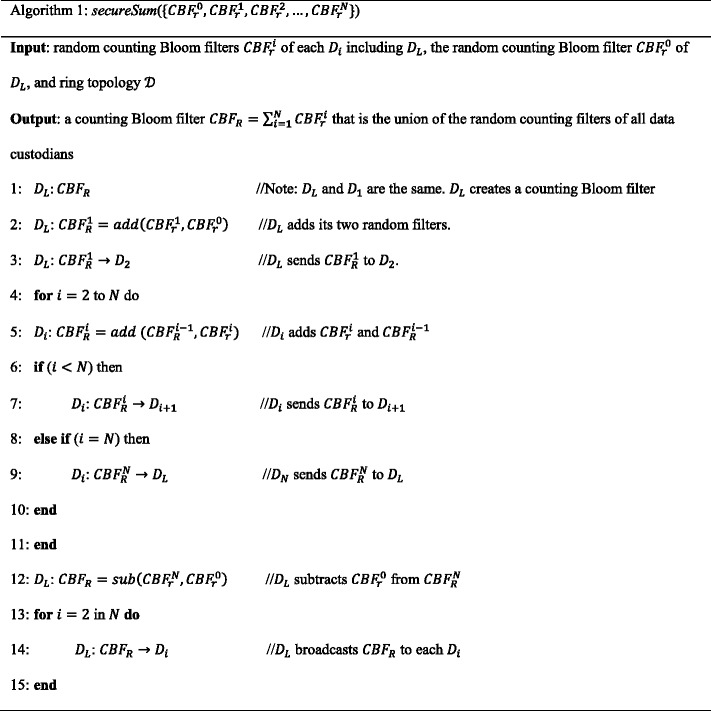


In steps 1–3, the leader data custodian *D*_*L*_ computes *CBF*_*R*_^1^ = *add*(*CBF*_*r*_^1^, *CBF*_*r*_^0^) and sends the result *CBF*_*R*_^1^ to data custodian *D*_2_. In steps 4–11, each data custodian *D*_*i*_, in turn, computes *CBF*_*R*_^*i*^ = *add*(*CBF*_*r*_^*i*^, *CBF*_*R*_^*i* − 1^) where 2 ≤ *i* ≤ *N* and *CBF*_*R*_^*i* − 1^ is the value received from the previous data custodian *D*_*i* − 1_. Then, *D*_*N*_ sends its result *CBF*_*R*_^*N*^ to *D*_*L*_. In step 12, *D*_*L*_ computes *CBF*_*R*_ = *sub*(*CBF*_*r*_^*N*^, *CBF*_*r*_^0^) and gets the actual sum $CB{F}_R={\displaystyle {\sum}_{i=1}^NCB{F}_r^i}$. In steps 13–15, *D*_*L*_ broadcasts *CBF*_*R*_ to all data custodians.

In this protocol, collusion between data custodians *D*_*i* − 1_ and *D*_*i* + 1_ reveals the secret value of *D*_*i*_. Extensions to the protocol are proposed in [21, 53] to make collusion between data custodians difficult.

## A secure deduplication protocol

In this section, we describe the secure deduplication protocol proposed in this paper. The protocol includes the setup and computation phases.

### Setup phase

**Fig. 4:**
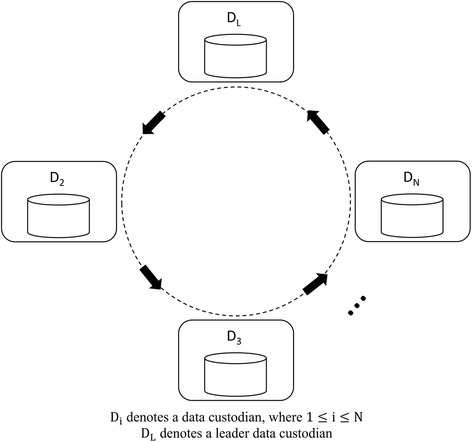
Ring topology of the data custodians

In this phase, the *coordinator* broadcasts a start message that contains the user query criteria and the *P*(*false positive*) value to each *D*_*i*_ in $\mathcal{D}$. Then, the data custodians jointly select the leader data custodian, denoted as *D*_*L*_. For simplicity, let us assume that *D*_1_ is selected as the leader. Then, they form a ring topology, *D*_*L*_ → *D*_2_ → *D*_3_ → … → *D*_*i*_ → *D*_*i* + 1_ → … → *D*_*N*_, as shown in Fig. 4.

The data custodians jointly select the required parameters, such as the expected number of records *n*, Bloom filter size *m*, number of hash functions *k*, counter size *c*, and *P*(*false positive*). The data custodians also agree on a cryptographic hash function *H*_0_(.) and the *k* hash functions *H*_*k*_ with two secret keys *k*_0_ and *k*_1_.

### Computation phase

The computation phase contains two subprotocols, such as the secure duplicate identifier and the distributed sorted neighborhood. The secure duplicate identifier subprotocol allows each data custodian to learn which of its records have duplicate records in the VD with false positive probability *P*(*false positive*). Then, the distributed sorted neighborhood subprotocol is executed on the results of the secure duplicate identifier subprotocol to identify the real duplicate records and remove the duplicate records while maintaining a single occurrence of the records.

#### A secure duplicate identifier subprotocol

The objective of this subprotocol is to allow each data custodian *D*_*i*_ to identify which of its records has a duplicate in the VD with a small false positive probability *P*(*false positive*). The protocol consists of the following steps:

1. Each *D*_*i*_ in $\mathcal{D}$ performs the following steps:
  - Extract from its local dataset a set of unique IDs, denoted as *I*_*i*_, of the patients who satisfy the user query criteria.
  - Encode *I*_*i*_ as the counting Bloom filter *CBF*_*r*_^*i*^ using the keyed hash functions *H*_*k*_ with the secret key *k*_1_.
  - Create the random counting Bloom filter *CBF*_*r*_^*i*^ (the algorithm used to create the random counting Bloom filter is described in Additional file 1).
2. *D*_*L*_ creates the initial random counting Bloom filter *CBF*_*r*_^0^.
3. The data custodians $\mathcal{D}$ jointly run Algorithm 1 to compute the sum $CB{F}_R={\displaystyle {\sum}_{i=1}^NCB{F}_r^i}$ .
4. Each *D*_*i*_ sums *CBF*_*r*_^*i*^ and *CBF*_*I*_^*i*^ and sends the result *CBF*_*r* ∪ *I*_^*i*^ to the *coordinator*.
5. The *coordinator* computes the sum $CB{F}_{R\cup S}={\displaystyle {\sum}_{i=1}^N}$ *CBF*_*r* ∪ *I*_^*i*^.
6. For each *D*_*i*_ in $\mathcal{D}$, the *coordinator* performs the following steps:
  - Convert *CBF*_*r* ∪ *I*_^*i*^ into the Bloom filter *BF*_*r* ∪ *I*_^*i*^.
  - Intersect *CBF*_*R* ∪ *S*_ and *BF*_*r* ∪ *I*_^*i*^ and send the result *CBF*_*R* ∪ *S*_ ∩ *BF*_*r* ∪ *I*_^*i*^ to *D*_*i*_.
7. Each *D*_*i*_ in $\mathcal{D}$ performs the following steps:
  - Create the Bloom filters *BF*_*I*_^*i*^ and *BF*_*r* ∪ *I*_^*i*^ from the counting Bloom filters *CBF*_*I*_^*i*^ and *CBF*_*r* ∪ *I*_^*i*^, respectively.
  - Intersect *CBF*_*R*_ and *BF*_*r* ∪ *I*_^*i*^ and create the counting Bloom filter *CBF*_*R*_ ∩ *BF*_*r* ∪ *I*_^*i*^.
  - Subtract *CBF*_*R*_ ∩ *BF*_*r* ∪ *I*_^*i*^ from *CBF*_*R* ∪ *S*_ ∩ *BF*_*r* ∪ *I*_^*i*^. The result is denoted by (*CBF*_*R* ∪ *S*_ ∩ *BF*_*r* ∪ *I*_^*i*^) − (*CBF*_*R*_ ∩ *BF*_*r* ∪ *I*_^*i*^) = (*CBF*_*R* ∪ *S*_ − *CBF*_*R*_) ∩ *BF*_*r* ∪ *I*_^*i*^. However, we know that *CBF*_*R* ∪ *S*_ − *CBF*_*R*_ = *CBF*_*S*_. Therefore, the result can be expressed by *CBF*_*S*_ ∩ *BF*_*r* ∪ *I*_^*i*^.
  - Intersect *CBF*_*S*_ ∩ *BF*_*r* ∪ *I*_^*i*^ and *BF*_*I*_^*i*^ and create the counting Bloom filter denoted as *CBF*_*S*_ ∩ *BF*_*r* ∪ *I*_^*i*^ ∩ *BF*_*I*_^*i*^. The expression can be reduced to *CBF*_*S* ∩ *I*_^*i*^ = *CBF*_*S*_ ∩ *BF*_*I*_^*i*^, as *BF*_*r* ∪ *I*_^*i*^ ∩ *BF*_*I*_^*i*^ is equal to *BF*_*I*_^*i*^.
  - Query the number of occurrences of the IDs *I*_*i*_ in *CBF*_*S* ∩ *I*_^*i*^ using the *count*() function, and create the list *L*_*i*_ that contains the IDs that have more than one occurrence.

In steps 1–2, each data custodian *D*_*i*_ (where 1 ≤ *i* ≤ *N*) encodes the unique IDs of its records as the counting Bloom filter *CBF*_*I*_^*i*^ and creates the random counting Bloom filter *CBF*_*r*_^*i*^. The leader data custodian *D*_*L*_ creates the additional random counting Bloom filter *CBF*_*r*_^0^. In step 3, the data custodians jointly compute the sum of their random counting Bloom filters, $CB{F}_R={\displaystyle {\sum}_{i=1}^NCB{F}_r^i}$, using Algorithm 1. In step 4, *D*_*i*_ computes *CBF*_*r* ∪ *I*_^*i*^ = *add*(*CBF*_*r*_^*i*^, *CBF*_*I*_^*i*^) and sends the result *CBF*_*r* ∪ *I*_^*i*^ to the *coordinator*. In step 5, the *coordinator* sums all data custodians’ *CBF*_*r* ∪ *I*_^*i*^, $CB{F}_{R\cup S}={\displaystyle {\sum}_{i=1}^N}$ *CBF*_*r* ∪ *I*_^*i*^.

In step 6, the *coordinator* computes *BF*_*r* ∪ *I*_^*i*^ = *toBloomFilter*(*CBF*_*r* ∪ *I*_^*i*^) and *CBF*_*R* ∪ *S*_ ∩ *BF*_*r* ∪ *I*_^*i*^. The *coordinator* sends *CBF*_*R* ∪ *S*_ ∩ *BF*_*r* ∪ *I*_^*i*^ to *D*_*i*_. In step 7, each data custodian *D*_*i*_ creates the counting Bloom filter *CBF*_*S* ∩ *I*_^*i*^ = *CBF*_*S*_ ∩ *BF*_*I*_^*i*^ that encodes the intersection between the IDs of *D*_*i*_ and all data custodians. Finally, *D*_*i*_ queries its IDs in *CBF*_*S* ∩ *I*_^*i*^ to create the list *L*_*i*_ that contains the IDs for the records that are likely to be duplicates with the false positive probability *P*(*false positive*). Although the *P*(*false positive*) is very small, for some applications it may not be acceptable, and the true duplicate records should be identified.

The total number of likely duplicate records across the data custodians, ${\displaystyle {\sum}_{i=1}^N\left|{L}_i\right|}$ is very small compared to the total number of records, as the number of records that have duplicate records is often a small proportion of the total number of records. Therefore, we can run existing deterministic PPRL protocols [12, 22, 24, 27] on the results of the secure duplicate identifier subprotocol with minimal computation and communication complexity. In the next section, we present an improved protocol based on the keyed hash function that reduces the required number of comparisons.

#### Secure distributed sorted neighborhood subprotocol

In the conventional sorted neighborhood (SN) technique [54, 55], sorting keys are generated for each record using a single attribute or a concatenation of the attributes of the records, and the keys are sorted lexicographically. Then, a sliding window of fixed size *w* is moved over the sorted records, and only the records in the same window are compared.

After the secure duplicate identifier subprotocol is run, each data custodian *D*_*i*_ has the list *L*_*i*_ that contains the IDs of the likely duplicate records. Note that the size of *L*_*i*_ is much smaller than the total number of records of *D*_*i*_. In a simple approach for finding the actual duplicate records, *D*_*i*_ hashes each ID in *L*_*i*_ using a keyed hash function and sends the result *HL*_*i*_ to the *coordinator*, who computes the union of the hash lists from all data custodians, *HL* = ⊎_*i*_ *HL*_*i*_. Then, the coordinator performs exact matching between every ID with every ID in *HL*. However, in practice, we know that most of the comparisons are unlikely to match.

Let us assume that a set of data custodians ${\mathcal{D}}_s\subseteq \mathcal{D}$ and each data custodian ${D}_i\in {\mathcal{D}}_s$ has the record *r*_*j*_ with the ID *j*. As *r*_*j*_ is a duplicate record, value *j* appears in the list of IDs of the likely duplicate records, *L*_*i*_, of each ${D}_i\in {\mathcal{D}}_s$. *H*_0_(*j*) denotes the hash of *j* with hash function *H*_0_(.). As the hash of *j* with the same hash function multiple times gives the same hash values, each ${D}_i\in {\mathcal{D}}_s$ sends to the *coordinator* the list *HL*_*i*_ that contains *H*_0_(*j*) . Therefore, *HL* contains multiple occurrences of *H*_0_(*j*), and sorting *HL* brings hash values for the same ID next to each other.

Based on these observations, we present a distributed sorted neighborhood (DSN) subprotocol that extends the SN technique. The protocol parallelizes the sorting by making each data custodian *D*_*i*_ locally sort *HL*_*i*_, and the *coordinator* merges only the sorted lists. The DSN protocol has the following steps:

1. Each *D*_*i*_ in $\mathcal{D}$ performs the following steps:
  - For every ID *j* in *L*_*i*_, *D*_*i*_ performs the following steps:
    1. Hash *j* using the keyed hash function *H*_0_(.) with the secret key *k*_0_.
    2. Store the hash of *j* in the list *SL*_*i*_.
  - Lexicographically sort *SL*_*i*_.
  - Send *SL*_*i*_ to the *coordinator*.
2. The *coordinator* performs the following steps:
  - Merge the *SL*_*i*_ of each *D*_*i*_ in $\mathcal{D}$ and create the list *SL*.
  - Slide a window of size *w* over the list *SL* and compare each pair of hash IDs within the window. If at least two hash IDs match, then the records associated with the IDs are duplicates.
  - Send to *D*_*i*_ the list *DL*_*i*_ of the hash IDs of the records that *D*_*i*_ needs to remove from its local dataset.
3. Each *D*_*i*_ in $\mathcal{D}$, for every ID *j* in *DL*_*i*_, removes its record associated with *j*.

### Extension of the secure deduplication protocol for deterministic algorithms

Thus far, the proposed protocol has been described for situations in which a common unique identifier exists, which enables efficient and high-quality linkage. This assumption is realistic in countries, such as Norway, Sweden, and Denmark, where a high-quality unique personal identifier is available [56, 57].

However, in many situations, the available unique identifier is low quality or does not exist. We describe how our protocol can be extended to support deterministic record linkage algorithms that define the criteria about which identifiers need to match in order to accept the linkage of a pair of records.

To increase the quality of the linkage, data cleaning often precedes record linkage. We also assume appropriate data cleaning occurs before the protocol is run. Various data-cleaning techniques, such phonetic encoding algorithms, have been proposed in the literature [58].

It has been shown that a linkage key can be created based on a concatenation of quasi-identifiers, such as name, sex, date of birth, and address. Studies have estimated that up to 87% of the U.S. population [59], 98% of the Canadian population [60], and 99% of the Dutch population [61] are unique, with a combination of quasi-identifiers, such as postal code, sex, and date of birth.

The National Cancer Institute in the United States uses a deterministic record linkage algorithm to link Surveillance, Epidemiology and End Results (SEER) data collected from cancer registries and Medicare claims data. The algorithm creates linkage keys using a set of criteria based on a Social Security number (SSN), first name, last name, date of birth, and sex [62, 63].

Let us consider a deterministic record linkage algorithm that has *p* linkage keys where each linkage is generated using a distinct match criterion defined by combinations of quasi-identifiers. For each match criterion, each data custodian creates a linkage key, and the deduplication protocol is run with the linkage key the same way the protocol is run with a unique identifier. However, in the distributed sorted neighborhood subprotocol, each data custodian sends the hash of the local identifiers of the likely duplicate records with the hash of the linkage keys to the *coordinator*. Finally, the *coordinator* identifies the actual duplicate records from the results of the protocol with all the linkage keys.

Let us consider, for simplicity of description, that each data custodian has an equal number of records. The computation time for a data custodian to create linkage keys for its records based on a combination of quasi-identifiers is denoted as *t*_*l*_. The runtime for the protocol using a unique identifier is denoted as *t*_*u*_. Assuming that the data custodians generate linkage keys for their records in parallel, deduplication using a linkage key has a total runtime of *t*_*u*_ + *t*_*d*_.

For a deterministic record linkage algorithm that has *p* linkage keys, the total runtime is *p* × (*t*_*u*_ + *t*_*d*_) + *t*_*a*_, where *t*_*a*_ is the sum of the additional time required to send local unique identifiers to the *coordinator* and the computation time for the *coordinator* to find the actual duplicate by combining the results of the protocol with each linkage key. However, as a separate instance of the protocol can run with each linkage key in parallel, the runtime reduces to (*t*_*u*_ + *t*_*d*_) + *t*_*a*_.

## Results

In this section, we describe the security analysis and the implementation of the proposed deduplication protocol. We also describe the theoretical and experimental evaluations of the protocol’s efficiency and scalability.

### Security analysis

We prove the security of the proposed protocol in the presence of corrupt data custodians or a corrupt *coordinator* who tries to learn information as a result of the protocol execution. We assume that a corrupt *coordinator* does not collude with a corrupt data custodian.

For the security proof of the protocol, we follow the standard security definition that is called privacy by simulation. For an adversary that controls a set of data custodians (or the *coordinator*), the adversary’s view (the information learned during the protocol execution) is a combination of the corrupt data custodians’ views. The adversary also accesses the corrupt data custodians’ inputs and outputs. Thus, in the simulation paradigm, we require to show the existence of an efficient simulator that can generate the adversary’s view in the protocol execution given only the corrupt data custodians’ inputs and outputs.

#### **Theorem 1 (compromised D**_**i**_**)**

*The protocol is secure against an honest-but-curious adversary ADV that corrupts (or controls) at most N* − 2 *data custodians*.

#### *Proof*

We prove the robustness of the protocol by looking at the exchanged messages and reducing the security to the properties of the Bloom filter.

We denote the corrupt data custodians as ${\mathcal{D}}_A\subset \mathcal{D}$, where $\left|{\mathcal{D}}_A\right|\le N-2$. The inputs to a simulator are the inputs and outputs of the corrupt data custodians ${\mathcal{D}}_A$. The inputs and outputs of each corrupt data custodian ${D}_a\in {\mathcal{D}}_A$ are the list of the IDs of its records *I*_*a*_ and the list of the IDs for likely duplicate records *L*_*a*_, respectively.

The view of each corrupt data custodian ${D}_a\in {\mathcal{D}}_A$ are the counting Bloom filters, such as *CBF*_*I*_^*a*^, *CBF*_*S* ∩ *I*_^*a*^, *CBF*_*r*_^*a*^ and *CBF*_*R*_. *CBF*_*I*_^*a*^ and *CBF*_*S* ∩ *I*_^*a*^ can be generated from lists *I*_*a*_ and *L*_*a*_, respectively. *CBF*_*r*_^*a*^ and *CBF*_*R*_ are randomly generated. In general, the simulator can generate the adversary’s view in the protocol execution from the corrupt data custodians’ inputs and outputs. Thus, the protocol is secure against an honest-but-curious adversary so that the protocol computes without revealing anything except the outputs. Therefore, the adversary cannot extract any private information about patients who have records at honest data custodians. In addition, the adversary cannot learn the number of records at honest data custodians.

Let us assume that the ID *j* for a duplicate record *r*_*j*_ is in the list *L*_*a*_ of corrupt data custodian ${D}_a\in {\mathcal{D}}_A$. The adversary learns the number of duplicates for *r*_*j*_ from *CBF*_*S* ∩ *I*_^*a*^ with a false-positive probability equal to *P*(*false positive*), denoted as *d*. The adversary can look into its inputs to learn the actual number of duplicates of *r*_*j*_ at ${\mathcal{D}}_A$, denoted as *d*_*A*_. Therefore, an adversary may infer whether duplicate records for *r*_*j*_ exist at honest data custodians with the following probability:

$$ p=\raisebox{1ex}{$\left(d-{d}_A\right)\left(1-P\left( false\  positive\right)\right)$}\!\left/ \!\raisebox{-1ex}{$\left(N-\left|{\mathcal{D}}_A\right|\right)$}\right.. $$

#### **Theorem 2 (compromised coordinator)**

*An honest-but-curious adversary ADV that corrupts the coordinator cannot infer any information about the presence or absence of patients at data custodians and the number of records contributed by a data custodian*.

#### *Proof*

We prove the security of the protocol by analyzing the messages received by the *coordinator* during the execution of the protocol and reduce its security to the properties of the hash functions *H*_*k*_ and *H*_0_(.) used in the protocol.

The *coordinator’s* view is the counting Bloom filter *CBF*_*r* ∪ *I*_^*i*^ and the list of the hash IDs of the likely duplicate records *SL*_*i*_ of each data custodian *D*_*i*_. The *coordinator* does not have inputs and outputs. The objective of the security proof is to show that private information about patients and data custodians cannot be learned based on the *coordinator’s* view during the protocol execution.

The secret keys (*k*_0_, *k*_1_) used by the data custodians are not available to the simulator. Therefore, the simulator cannot learn the IDs of the records inserted in *CBF*_*r* ∪ *I*_^*i*^. In addition, as the hash function *H*_0_(.) is cryptographically secure, the simulator cannot learn the IDs based on *SL*_*i*_.

Each array position of *CBF*_*r* ∪ *I*_^*i*^ has a counter value equal to the sum of the corresponding counter values of *CBF*_*I*_^*i*^ and *CBF*_*r*_^*i*^. Thus, the counter values of *CBF*_*r* ∪ *I*_^*i*^ are random, as every counter position of *CBF*_*r*_^*i*^ has a random value. The random noise *CBF*_*r*_^*i*^ inserted in *CBF*_*r* ∪ *I*_^*i*^ prevents the simulator from learning the approximate total number of records of *D*_*i*_ encoded by *CBF*_*I*_^*i*^.

Therefore, *ADV* cannot learn the IDs and the number of records held at a data custodian, and consequently, the protocol is secure in the face of a corrupt *coordinator*.

### Implementation

A prototype of the proposed deduplication protocol is implemented in Java. The prototype contains the local and *coordinator* software components. The local software component is deployed at each data custodian, while the *coordinator* software component is deployed at the *coordinator*. The parameters required for an instance of the protocol are configured through the configuration files.

The dataset at each data custodian was stored in a MySQL relational database. We used the JavaScript Object Notation (JSON) format for message communication. Each component used an Extensible Messaging and Presence Protocol (XMPP) [64] client to connect to an XMPP server. Then, a JSON message was sent through the XMPP server between two entities that participate in the protocol. All messages were compressed using the Lz4 [65] lossless compression algorithm to reduce the overall size. After transmission, each message was decompressed before actual use.

### Analytical evaluation

The main concerns for the practical use of SMC protocols are efficiency and scalability. Efficiency is the ability to compute with a good performance, which is often expressed by the communication and computation complexity. Communication complexity is analyzed in terms of the communication rounds and the size of the messages exchanged between the parties involved in the protocol. For *N* data custodians, each data custodian sends three messages and receives three messages, except the leader data custodian that sends *N* + 2 messages. The *coordinator* sends 2*N* messages and receives 2*N* messages. The overall communications of the protocol is 6*N* − 1, which is linear with the number of data custodians *O*(*N*).

The size of a message that contains a counting Bloom filter depends on the Bloom filter size *m* and counter size *c*. The size of the message that contains the list of likely duplicate hash IDs *SL*_*i*_ is small compared to the size of the message that contains the IDs *I*_*i*_ of all records, but it depends on the false positive probability and the proportion of the records of *D*_*i*_ that have duplicates in the virtual dataset.

Computation complexity is measured in terms of the time it takes for each entity to complete local computations and the protocol runtime. Scalability is measured in terms of the change in efficiency as the number of records and data custodians increases.

In general, the local computations of the protocol are computationally very efficient, as it does not use a building block that adds overhead to the performance. Bloom filter operations require only *O*(1) time. The other computations are performed only on the list of the hash IDs of the likely duplicate records, which are a small proportion of the IDs of all the records. In addition, the data custodians often compute in parallel. Detailed analysis of the total computation time is provided in Additional file 1.

### Experimental evaluation

We ran the experiments using actual and simulated datasets to evaluate the efficiency and scalability of the protocol. The experiments were run 100 times, and the average total runtime for the protocol and the local computation time for each entity were recorded. In this section, we report only the total runtimes. Details regarding the parameters used for the experiments are given in Additional file 1.

#### In situ experiments

We deployed the prototype at three microbiology laboratories located in Norway on top of the Snow disease surveillance system [36]. Two laboratories are departments at the University Hospital of North Norway (UNN) and Nordland Hospital (NLSH). The third laboratory is Fürst Medical Laboratory, a private laboratory. UNN and NLSH together cover a total population of more than 475 000 inhabitants. Based on the number of laboratory tests conducted within a specific time period, we estimated that the Fürst population coverage is approximately twice the total population covered by UNN and NLSH.

At Fürst, the local software component was deployed on an Intel i5-4590 3.3GHz quad core with 8GB RAM and Ubuntu 14.04. At UNN and NLSH, the local software component was deployed on an Intel Xeon E5-2698 v3 2.3GHz dual core with 4GB RAM and Red Hat Enterprise Linux 7. The coordinator software component was deployed on an Intel Xeon X3220 2.4GHz quad core with 8GB RAM and Ubuntu 14.04. The laboratories and the *coordinator* were connected through the Norwegian Health Network, a wide area network of healthcare service providers. Details about the network connections and the communication patterns are described in Additional file 1.

We ran two experiments on the data distributed across the three laboratories. The first experiment involved answering a query about the number of individuals infected with influenza A between January 2015 and April 2016. Each laboratory locally queried the IDs of the individuals who were tested positive for influenza A during this time period, and a virtual dataset that contained 5329 records was created.

The second experiment involved answering a query about the number of patients who were tested at multiple laboratories between January 2015 and April 2016. Each laboratory locally queried the unique IDs of the patients who had been tested for any of the diseases included in the Snow system during the time period, and a virtual dataset that contained 85 353 unique IDs was created.

We divided each virtual dataset into segments by varying the time period in which the analyses were performed. Then, we ran the protocol on each segment of the virtual datasets.

**Fig. 5:**
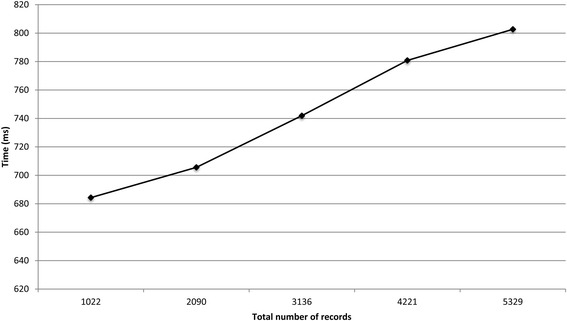
The total runtime for the protocol on the influenza A datasets

**Fig. 6:**
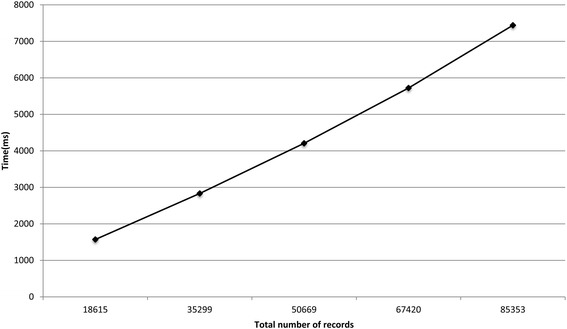
The total runtime for the protocol on the datasets that contain the test results for various diseases

Figures 5 and 6 show the runtimes for the protocol on the two virtual datasets as the total number of records increased. The deduplication of 5329 and 85 353 records took around 0.8 and 7.5 s, respectively. The local computation time for the laboratories and the *coordinator* is presented in Additional file 1. For the deduplication of 85 353 records, the local computation time for the *coordinator* and the data custodians did not exceed 0.6 s.

The experiment on the VD of the positive test results for influenza A found one patient who was tested at multiple laboratories between January 2015 and April 2016. The experiment on the VD that contained the test results for various diseases found 449 patients who had been tested at multiple laboratories for different infectious diseases. The results showed that the samples collected from each patient were tested at multiple laboratories at different times.

#### In vitro experiments

We deployed the prototype of the protocol on a cluster computer. Each node had two Intel E8500 dual-core 1.17GHz CPUs, 4GB RAM, and CentOS 6.7. The nodes were connected through fast Ethernet.

We ran experiments on simulated virtual datasets that consisted of a large number of data custodians and records. The VDs consisted of a varying number of data custodians (i.e., 5, 10, 15, and 20) and total number of records (i.e., 200 000, 400 000, 600 000, 800 000, and 1 000 000). The total number of records of each VD was distributed equally among all the data custodians, and each data custodian contained around 5% duplicate records. Details about the datasets are provided in Additional files 1 and 2.

**Fig. 7:**
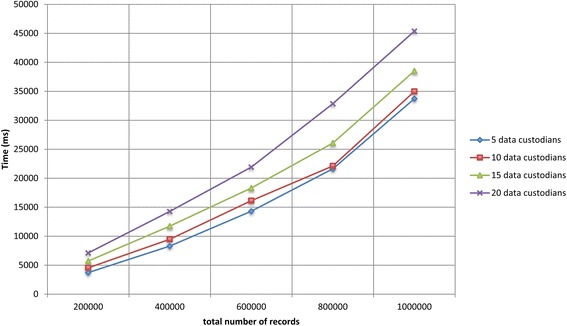
The total runtime for the protocol on the simulated datasets as the total number of records increases

**Fig. 8:**
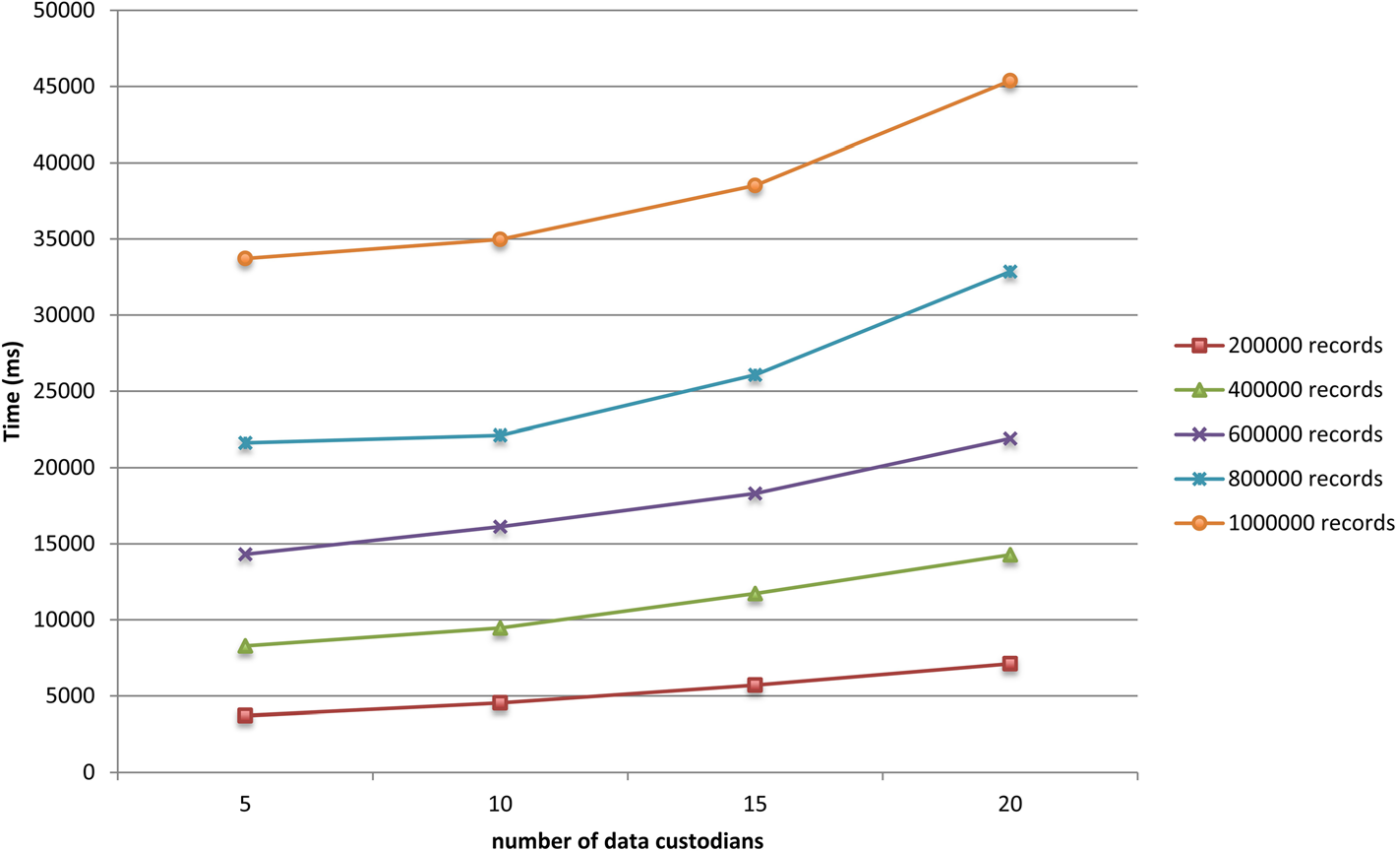
The total runtime for the protocol on the simulated datasets as the number of participating data custodians increases

Figures 7 and 8 show the total runtime for the protocol as the total number of records in the virtual dataset and the number of participating data custodians increased, respectively. The deduplication of one million records distributed across five and 20 data custodians took around 34 and 45 s, respectively. The local computation time for the laboratories and the *coordinator* is presented in Additional file 1. In general, the local computation time for the *coordinator* and the data custodians did not exceed 34 and 7 s, respectively.

## Discussion

We proposed a privacy-preserving protocol for the deduplication of data horizontally partitioned across multiple data custodians. The protocol protects the privacy of patients and data custodians under the semi-honest adversarial model. The protocol remains secure even when up to *N* − 2 data custodians collude.

The protocol satisfies the security requirements that were formulated in [26] for a secure record linkage protocol. However, we assumed that the *coordinator* has no means of getting the secret keys used in the protocol, which improves the efficiency and scalability of the protocol. The assumption can be ensured through a data use agreement among the data custodians that prohibits them from sharing the secret keys with the *coordinator*.

The protocol was deployed and evaluated in situ for the deduplication of test results distributed across three microbiology laboratories. The protocol was also evaluated in vitro on simulated microbiology datasets of up to one million records and 20 data custodians. The deduplication of the one million simulated records distributed across 20 data custodians was completed within 45 s. The experimental results showed that the proposed protocol is more efficient and scalable than previous protocols [29, 31].

The local computation time for the entities and the total runtime for the protocol scale linearly as the number of participating data custodians and the total number of records increase. The protocol scales because Bloom filter operations are not expensive, and the *coordinator* and data custodians perform most of the computations in parallel. In addition, the number of communication rounds of a data custodian is constant and does not increase with the addition of new data custodians.

The protocol was not experimentally evaluated for situations in which there is no unique identifier. However, we theoretically showed that the protocol remains scalable. The computation complexity of each party linearly increases with the number of steps of the deterministic record linkage algorithm.

There is a need for reuse health data at the regional, national, and global levels to increase the number of records and participating data custodians for a given study [66, 67]. The blocking technique [68] can be applied to increase the scalability of the proposed deduplication protocol to such a large scale. The intuition for the use of the blocking technique is that running a separate instance of the protocol on a subset of records that are likely to match enables parallel computations. A very simple example is running different instances of the protocol on the records of female and male patients.

In practice, data custodians create blocking keys for their records based on one or more attributes, and records that have the same blocking key values are grouped into the same block, which consequently divides the virtual dataset into segments. Then, the data custodians jointly execute a separate instance of the protocol for each segment of the virtual dataset in parallel. The data custodians can execute the protocol instances on different CPU cores or servers to increase the scalability of the protocol.

## Conclusions

Deduplication is a necessary preprocessing step for privacy-preserving distributed statistical computation of horizontally partitioned data. However, deduplication should not reveal any private information about individuals and data custodians. We proposed a privacy-preserving protocol for the deduplication of a horizontally partitioned dataset.

Efficiency and scalability are the main challenges for practical uses of SMC protocols. The experimental evaluations of the proposed protocol demonstrated its feasibility for a wide range of practical uses.

As discussed in the Discussion section, we plan to parallelize the execution of the protocol using the blocking technique. Furthermore, we also plan to integrate the protocol with the privacy-preserving distributed statistical computation framework we developed [20].

## Additional files

Additional file 1: It contains a description of the Bloom filter set operations, the computation complexity analysis of the protocol, the algorithm for generating random Bloom filters, the parameters used in the experiments, the datasets used in the experiments, the network connection for the in situ experiments, and additional experiment results. (DOCX 6563 kb)
Additional file 2: It is a Bash script that implements the algorithm we used to generate the simulated microbiology datasets. The script is discussed in Additional file 1. (SH 9 kb)

## Abbreviations

EHR: Electronic Health Record
JSON: JavaScript Object Notation
PPRL: Privacy-Preserving Record Linkage
SMC: Secure Multi-Party Computation
VD: Virtual Dataset
XMPP: Extensible Messaging and Presence Protocol

## Footnotes

1 Commutative encryption is a form of encryption in which the order of the consecutive encryption and decryption of a value with different cryptographic keys does not affect the final result and no two values have the same encrypted value [69].

2 The protocols are different from the protocol proposed in [70, 71] for probabilistic record linkage. In Schnell et al.’s protocol [70], for each record, each identifier is encoded as a separate Bloom filter, whereas in Durham et al.’s protocol [71], to avoid frequency-based cryptanalysis, the set of identifiers of each record is encoded as a Bloom filter.

3 Secret sharing is a method by which a secret value is split into shares and a predefined number of shares are required to reconstruct the secret value [30].

4 OT is a method for two parties to exchange one of several values in which the sender is oblivious to which value is selected, while the receiver learns only the selected value [32].

5 The Count-Min sketch [72] is, similar to the counting Bloom filter (see the description of the counting Bloom filter in the Methods section), a space-efficient probabilistic data structure for encoding a set of elements that allows querying the frequencies of the occurrence of the inserted elements with some error.
