# Supplementary material for: Secure and scalable deduplication of horizontally partitioned health data for privacy-preserving distributed statistical computation
Source: BMC Med Inform Decis Mak. 2017 Jan 3;17:1. doi: 10.1186/s12911-016-0389-x (PMC5209873; doi:10.1186/s12911-016-0389-x)
Supplement: Additional file 1: — It contains a description of the Bloom filter set operations, the computation complexity analysis of the protocol, the algorithm for generating random Bloom filters, the parameters used in the experiments, the datasets used in the experiments, the network connection for the in situ experiments, and additional experiment results. (DOCX 6563 kb) [file 12911_2016_389_MOESM1_ESM.docx]

Additional file 1

# Set operations on Bloom filters

In this section, we describe functions for set operations on counting Bloom filters, such as union, subtraction, and intersection. The section also presents functions for counting the number of occurrences of an element in a counting Bloom filter and converting a counting Bloom filter into a Bloom filter.

*Algorithm 2* is a pseudocode for computing the union of $CBF_{r}^{i}$ and $CBF_{I}^{i}$. $CBF_{r\cup I}^{i}$ denotes the union of $CBF_{r}^{i}$ and $CBF_{I}^{i}$ where each counter of $CBF_{r\cup I}^{i}$ is the sum of the counters of $CBF_{r}^{i}$ and $CBF_{I}^{i}$.


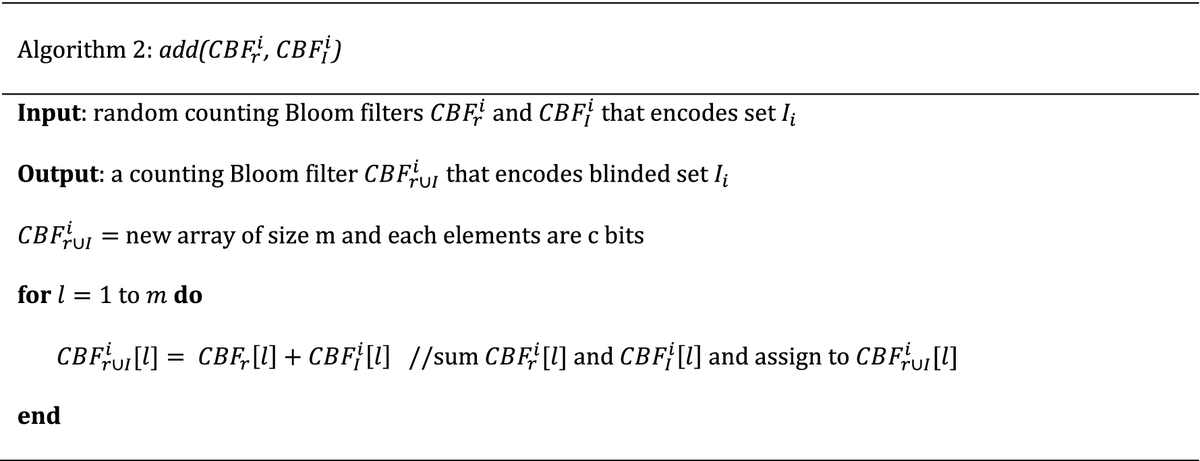


*Algorithm 3* is a pseudocode for subtracting ${CBF}_{R}$ from ${CBF}_{R\cup S}$. ${CBF}_{S}$ denotes the subtraction of ${CBF}_{R}$ from ${CBF}_{R\cup S}$ where each counter of ${CBF}_{S}$ is the subtraction of the counter of ${CBF}_{R}$ from the counter of ${CBF}_{R\cup S}$.


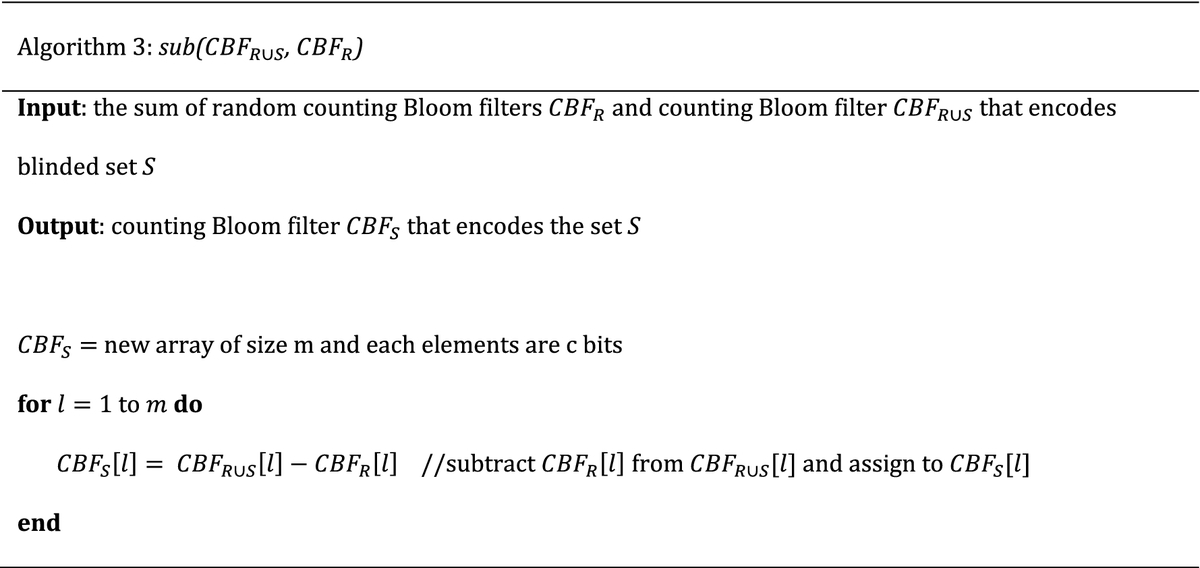


*Algorithm 4* is a pseudocode for creating a counting Bloom filter ${CBF}_{S\cap I}^{i}$ that encodes the intersection between sets $I_{i}$ and $S$ from ${CBF}_{S}$ and $BF_{I}^{i}$. The intuition of the algorithm is that for each element $x\in S\cap I_{i}$, the value of $BF_{I}^{i}$ is 1 at every position $b_{h}\left( x \right)$ and the value of ${CBF}_{S}$ is greater than or equal to 1. Similarly, for each element $x\notin S\cap I_{i}$, there is a high probability that the value of either $BF_{I}^{i}$ or ${CBF}_{S}$ is equal to zero at least at one of the positions $b_{h}(x)$.


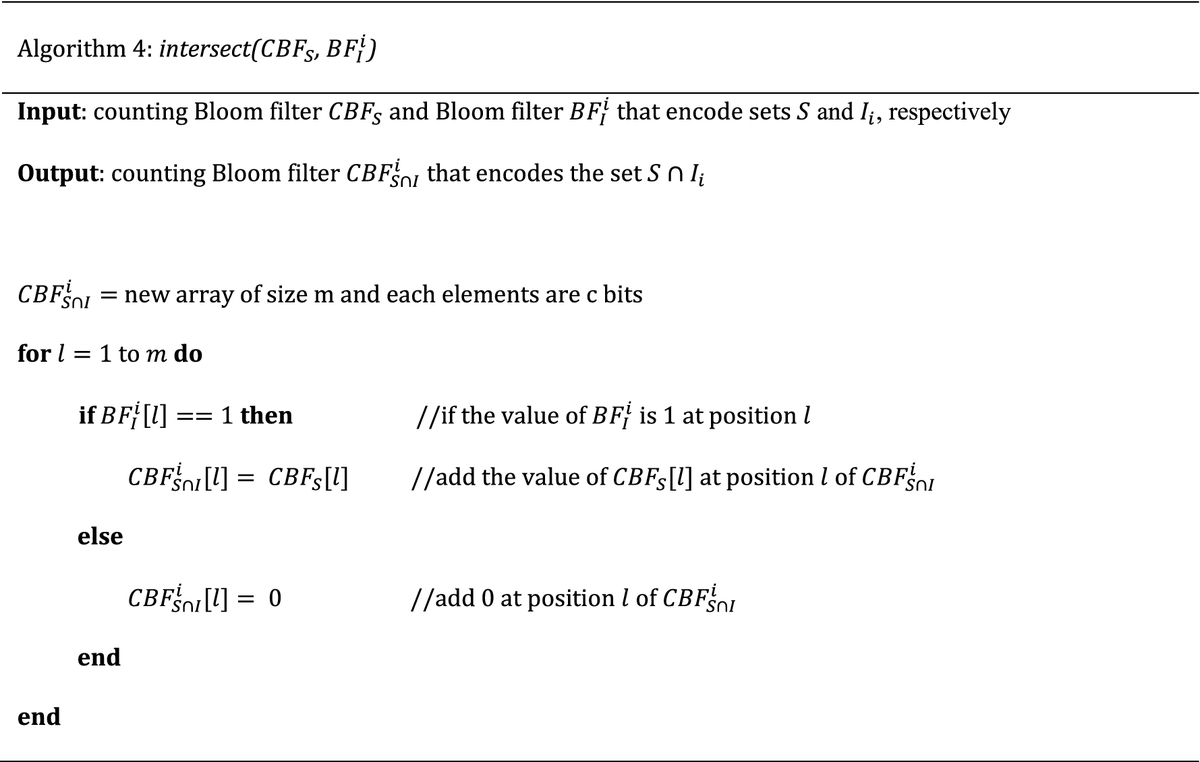


It is demonstrated in [1] that the minimum values of the counters at positions $b_{h}(x)$ is the count of $x$ in the counting Bloom filter with possible error equal to the false positive probability. *Algorithm 5* is a pseudocode for computing the number of occurrences of the element $x$ in ${CBF}_{S\cap I}^{i}$.


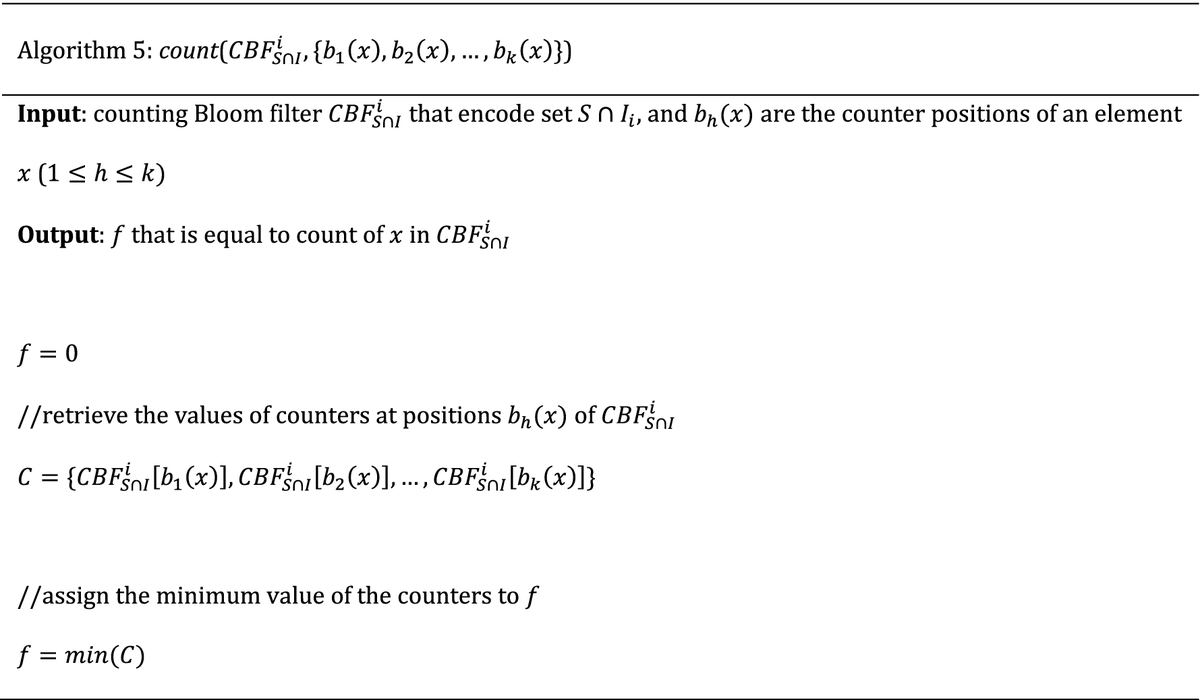


*Algorithm 6* is a pseudocode for converting the counting Bloom filter $CBF_{I}^{i}$ into the Bloom filter $BF_{I}^{i}$.


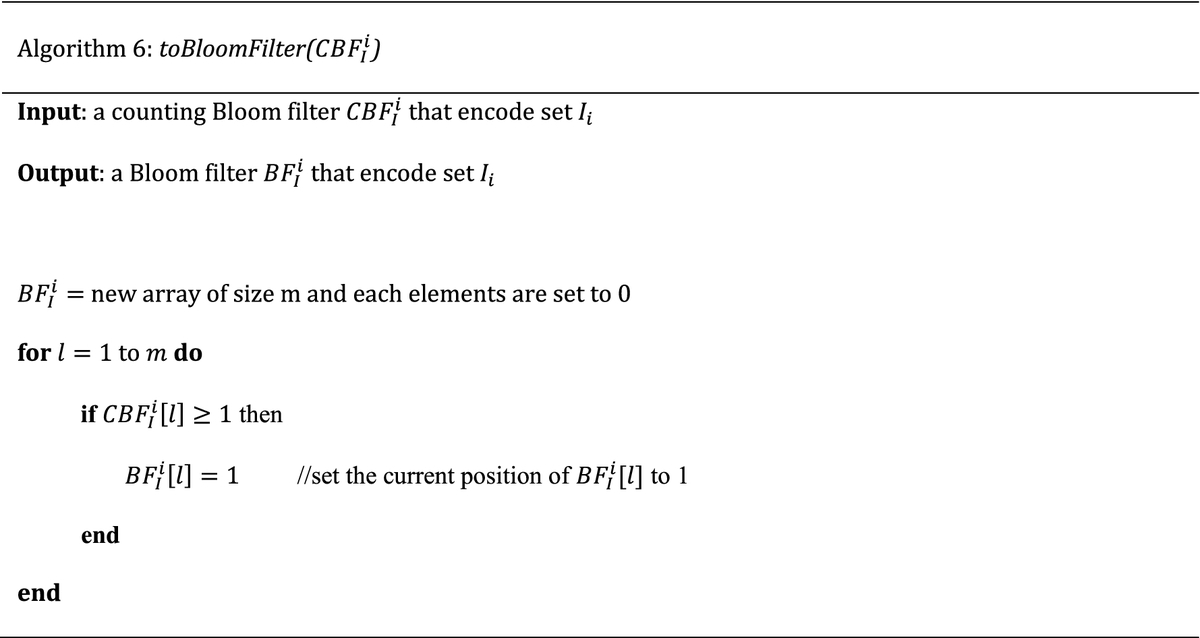


# Analytical evaluation of computation time

Let us consider, for simplicity of description, that each data custodian $D_{i}$ has an equal number of records $n_{i}$. The total number of records across the data custodians is $n=\sum_{i} n_{i}$.

Let $t_{o}$ be the time it takes to perform a single operation (i.e., insertion and membership query) on a Bloom filter or a counting Bloom filter. The total time to encode the list of unique IDs $I_{i}$ as the counting Bloom filter $CBF_{I}^{i}$ or to query $I_{i}$ in ${CBF}_{S\cap I}^{i}$ would be $n_{i}\cdot t_{o}$.

Let $t_{a}$ be the time it takes to perform a single arithmetic operation on two integer numbers. The union (addition) and subtraction operations are composed of arithmetic addition and subtraction operation on counter values at each array position of two counting Bloom filters, respectively. Intersection is composed of a comparison operation on counter values at each array position of a counting Bloom filter and a Bloom filter. Therefore, the total time for the union, subtraction, and intersection operations is approximately equal to $m\cdot t_{a}$, where $m$ is the Bloom filter length.

Let $t_{c}$ be the time it takes to execute the following function $f\left( x \right)=\left\{ \begin{aligned} 0, &x=0 \\ 1, &x\geq1 \end{aligned} \right.$, where $x$ is the counter value at each array position of a counting Bloom filter. The total time to convert a counting Bloom filter to a Bloom filter would be $m\cdot t_{c}$.

Let $t_{s}$ be the time it takes to execute *Algorithm 1.* In *Algorithm 1,* each data custodian performs the intersection operation on counting Bloom filters, and the leader data custodian also performs the subtraction operation on counting Bloom filters. Thus, $t_{s}$ can be expressed as $t_{s}=\left( N+1 \right)\cdot(m\cdot t_{a})$, where $N$ is the number of data custodians.

Let $t_{r}$ be the time it takes to create a random counting Bloom filter. Let $t_{h}$ be the time it takes to perform a single hash. The total computation time to get all the hash values for the list of IDs of the likely duplicate records $L_{i}$ would be $\left| L_{i} \right|\times t_{h}$. The time complexity of sorting the list of hash IDs of the likely duplicate records $HL_{i}$ using merge sort is $O(\left| L_{i} \right|\cdot\log\left| L_{i} \right|)$. Note that $\left| L_{i} \right|=\left| {HL}_{i} \right|$. The time complexity of merging the sorted list of hash IDs of the likely duplicate records $SL_{i}$ collected from $N$ data custodians is $O(\left| SL \right|)$, where $\left| SL \right|=\sum_{i} \left| SL_{i} \right|$.

Let $t_{sn}$ denotes the time it takes to slide a window with size $w$over the list $SL$ and compare every record pair. Let $t_{rm}$ denotes the time it takes for a data custodian to remove the duplicate records from its database.

In the steps where the data custodians compute in parallel, the computation time is the maximum time between the computation times of the data custodians. Therefore, the total computation time for the secure duplicate identifier subprotocol can be expressed with equation 1.

$\max_{i} (\left( n_{i}\cdot t_{o} \right)+t_{r})+ t_{r}+\left( N+1 \right)\left( m\cdot t_{a} \right)+\max_{i} (m\cdot t_{a})+\left( N-1 \right)\cdot\left( m .t_{a} \right)+\left( N\cdot\left( \left( m\cdot t_{c} \right)+\left( m\cdot t_{a} \right) \right) \right)+\max_{i} (2\cdot\left( m\cdot t_{c} \right)+3\cdot\left( m\cdot t_{a} \right)+\left( n_{i}\cdot t_{o} \right)),$ (1)

where $i\in[1,N]$. For simplicity of description, we assume that the data custodians have homogeneous computation resources, and consequently, the data custodians compute a given computation problem in equal time. Therefore, equation 1 can be expressed with equation 2.

$\max_{i} \left( 2 . (n_{i}\cdot t_{o}) \right)+ {2\cdot t}_{r}+\left( 3N+4 \right)\cdot\left( m\cdot t_{a} \right)+(N+2)\times\left( m\cdot t_{c} \right)$ (2)

Similarly, the total computation time for the secure distributed sorted neighborhood subprotocol can be expressed with equation 3.

$\max_{i} \left( \left| L_{i} \right|\cdot t_{h}+(\left| L_{i} \right|\cdot\log\left| L_{i} \right|) \right)+\left| SL \right|+t_{sn}+\max_{i} \left( t_{rm} \right)$(3)

The total computation time of the protocol is the sum of equation 2 and 3. As the computation times $t_{o}$, $t_{r}$, $t_{a},$ $t_{c}$, $t_{h}$, and $t_{rm}$ are very small and the values $\left| L_{i} \right|$ and $\left| SL \right|$ are much smaller than $n_{i}$ and $n$, respectively, the total computation time of the protocol is efficient.

# Random counting Bloom filter

In this section, we describe how a data custodian $D_{i}$ creates a random counting Bloom filter. As described in the paper, $CBF_{r}^{i}$ is an array of size $m$ and each counter has a size of $c$ bits. For now, let us redefine $CBF_{r}^{i}$ as a list of $m*c$ bits and initially all the bits are set to 0.

The data custodians agree on the maximum number of noise bits that can be set to 1 in $CBF_{r}^{i}$. However, we decided that the number of noise bits to be proportional to the number of records of the data custodian $D_{i}$. Therefore, the data custodians jointly choose a value for maximum noise percentile (between 0 and 1) denoted as $P$. Then, $D_{i}$ randomly choose its own private noise percentile $P_{i}<P$.

Let us denote the number of records of $D_{i}$ as $n_{i}$. Finally, $D_{i}$ randomly choose $P_{i}*n_{i}$ random integers between $0$ and $\left( m*c \right)-1$. Then, using the randomly chosen integers as input, it randomly sets the corresponding bit positions of $CBF_{r}^{i}$ to true or false. Let us denote each counter of $CBF_{r}^{i}$ as $C_{l}$ (where $1\leq l\leq m$). The way $CBF_{r}^{i}$ is created allows the insertion of a noise bit at any bit position of a counter $C_{l}$. Therefore, if $CBF_{r\cup I}^{i}$ is submitted to the coordinator, the coordinator cannot accurately estimate the number of records of $D_{i}$.

# Parameters used for the experiments

In this section, we describe the parameters used for the experiments that were performed in the paper.

It is demonstrated in [2] that the $k$ hash values of a Bloom filter can be effectively implemented with only two hash functions $H_{1}(x)$ and $H_{2}(x)$. The array positions $b_{h}(x)$ that correspond to the $k$ hash functions are simulated with the form $b_{h}(x)={(H}_{1}\left( x \right)+{h*H}_{2}(x)) mod m$ without affecting the false positive probability. A hash function $H(.)$ and two secret keys $k_{1}$ and $k_{2}$ can be used to instantiate the hash values of the two hash functions as $H_{1}(x)=H(k_{1}\parallel x)$ and $H_{2}(x)=H(k_{2}\parallel x)$. The hash function can be non-cryptographic hash function, which is often more efficient than cryptographic hash functions. For all the experiments, SHA-256 are used as $H_{0}(.)$. For the in situ experiments SHA-1 is used as $H_{k}$, whereas for the in vitro experiments we used MurmurHash 2.0 hash function.

In addition, a maximum noise percentile $P=0.05$ was used for all the experiments. As shown above, a small number of noise bits (that is proportional to the selected noise percentile) is sufficient to create a random counting Bloom filter $CBF_{r}^{i}$. Therefore, $CBF_{r}^{i}$ can sufficiently hide the count of records inserted into the private counting Bloom filter $CBF_{I}^{i}$ based on the blinded private counting Bloom filter $CBF_{r\cup I}^{i}$.

A counter size of four bits was used for all the experiments, except when the total number of records was equal to one million in which a counter size of five bits was used.

We selected a false positive probability $P \left( false positive \right)=0.1$. Note that the expected number of elements of a Bloom filter is denoted as $n$. In principle, the value of $n$ is equal to the sum of the number of records of all data custodians, $n=\sum_{i=1}^{N} n_{i}$, which can be securely computed using a secure summation protocol [3,4]. However, since the counting Bloom filter ${CBF}_{R\cup S}$ contains all the ids and random noises of all data custodians, the expected number of elements of ${CBF}_{R\cup S}$ is greater than $\sum_{i=1}^{N} n_{i}$. Since the maximum noise bits inserted were $5\%$ of $\sum_{i=1}^{N} n_{i}$, we decided that using an expected number of element value equal to $\sum_{i=1}^{N} n_{i}+0.2*\sum_{i=1}^{N} n_{i}$ is sufficient to keep the false positive probability to the minimum. For all Bloom filters used in the experiment the expected number of elements is computed as $\sum_{i=1}^{N} n_{i}+0.2*\sum_{i=1}^{N} n_{i}$.

Using the value of $n$ and $P(false positive)$ as input, the data custodians locally computed the optimal values for the required parameters, such as the Bloom filter size $m$ and the number of hash functions $k$ [5].

Since we assumed that no duplicate record exist at a single data custodian, the maximum number of possible duplicates of a record is equal to the number of participating data custodians $N$. Therefore, for the distributed sorted neighborhood subprotocol of all the experiments, we used the window size $w$ that is equal to the number of data custodians $N$.

# Datasets

The schema of the data extracted by the Snow system consists of a wide variety of attributes. The datasets used in this paper consist a subset of the attributes, such as unique patient identifier $j$, gender $g$, birth year $b$, municipality code $m$, infectious agent $a$, analysis date $t$, and test result $v$. The record of a patient is simply defined as $r(j,g, b, m, a,t, v)$.

We used two virtual datasets (VDs) consisting of 5329 and 85 353 records of actual microbiology laboratory tests results from the three microbiology laboratories in Norway.

We also used simulated VDs of microbiology laboratory test results created using a data generator script developed in this paper. The script is provided in the Additional file 2. The simulated VDs contain different number of records (i.e., 200 000, 400 000, 600 000, 800 000, and 1 000 000) distributed among data custodians (i.e., 5, 10, 15, and 20). The total number of records of each VD was distributed equally among all the data custodians, and each data custodian contained around 5% duplicate records.

The inputs to the data generator include the number of data custodians, total number of records, and percent of duplicate records. Let us consider a VD with five data custodians, 200 000 total number of records, and 5% duplicate records. As the total number of records in a VD is distributed equally among all the data custodians, each data custodian has 40 000 records. The number of unique and duplicate records of each data custodian is 38 000 and 2000 records, respectively.

First, the data generator script randomly creates the required number of unique records for each data custodian. Second, the duplicate records of a data custodian are generated by randomly copying records from the other data custodians.

The other input to the data generator script is a threshold value $r$ for each infectious agent. Two positive results of a patient for the same infectious disease are considered within the same episode when the difference between their analysis dates is $<r$ days, and otherwise the tests are considered within different episodes.

It is also possible to create duplicate records where two or more records of a patient are associated with distinct laboratory tests of an infectious agent with the same results. The duplicate records of a data custodian are created by randomly selecting patients who have a test results at the other data custodians, and creates similar record of each patient with a random analysis date. The analysis date is selected in such a way that the records within the same episode or different episodes with a given probability.

# Network connection for the in situ experiments

The network connections between the microbiology laboratories and the coordinator are as shown in Fig. 9. In Norway, healthcare service providers (i.e., general practitioners, hospitals, and medial laboratories) are connected to the Norwegian Health Network (HealthNet). The information and communications technology (ICT) systems of both UNN and NLSH are under the same administration of Health North ICT, which is the northern Norway regional health ICT. Therefore, the computation agents of UNN and NLSH are deployed on the same physical server but logically separated^[[1]](#footnote-1)^. The coordinator is also connected to the HealthNet through the same local area network as NLSH and UNN.


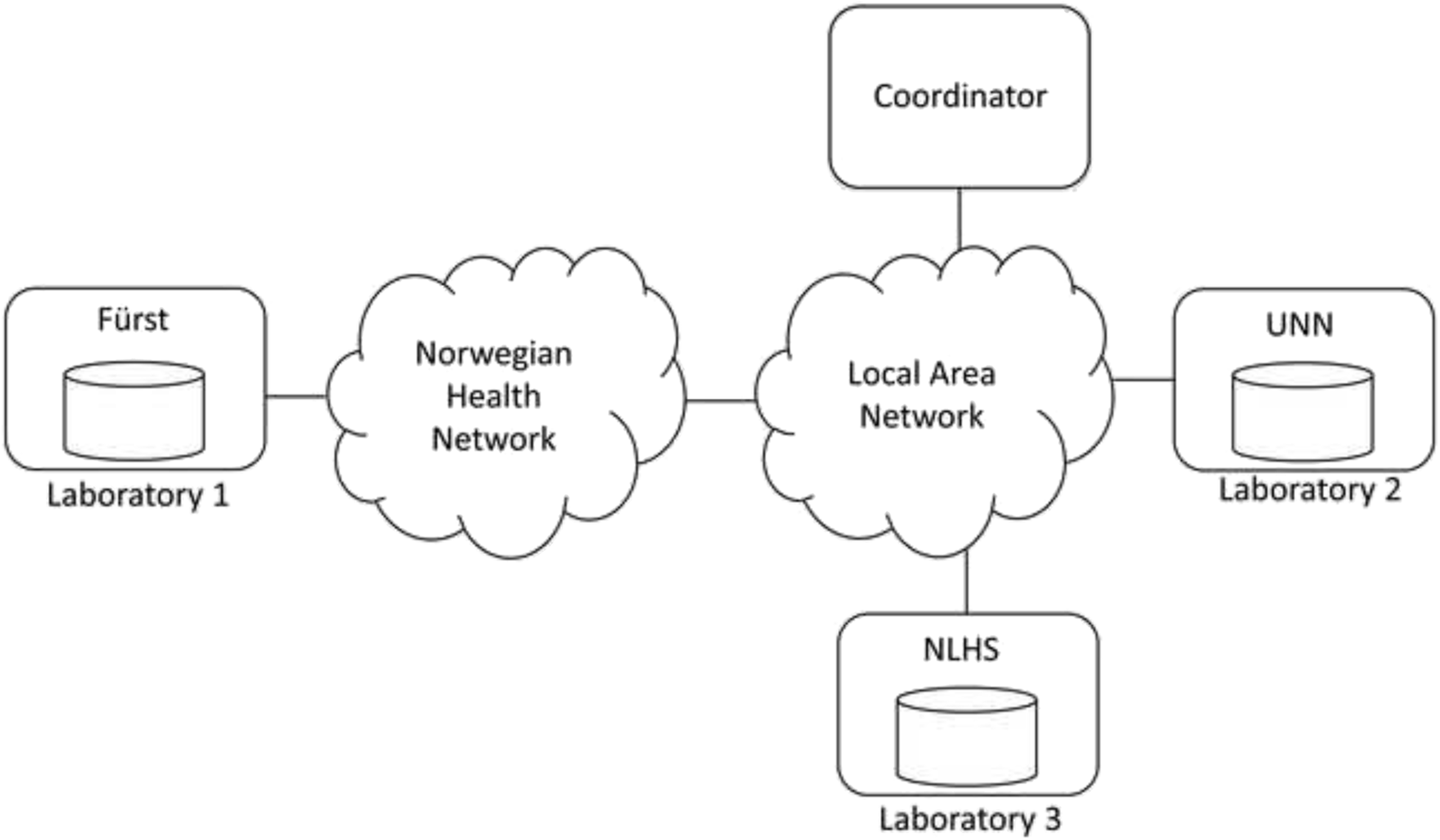


Fig. 9 A simplified view of the wide area network connections between the microbiology laboratories and the coordinator

The local area networks of the healthcare service providers are considered as high secure zone while the HealthNet is a low secure zone. Therefore, the coordinator is considered as low secure. All connections need to be initiated from high secure to low secure zone. Therefore, each laboratory initiates connection to the coordinator. Then, peer-to-peer communications between the laboratories are routed through the coordinator using end-to-end encryption. Even though UNN and NLSH are on the same server, their communications are always routed through the coordinator.

In all the experiments, Fürst is assigned as the leader data custodian that increases the number of communications over the HealthNet.

# Local computation time for the experiments

The time it took to query data from a database is excluded from the local computation times presented in this section.

## In situ experiments

In this section, we present the local computation times of the coordinator and the laboratories for the experiment ran on the virtual dataset containing 85,353 actual records of patients who had been tested for a set of diseases at UNN, NLSH, and Fürst medical laboratories between January 2015 and April 2016. Figure 10 shows the computation time of the *coordinator* to perform its local computations as the total number of records increases.


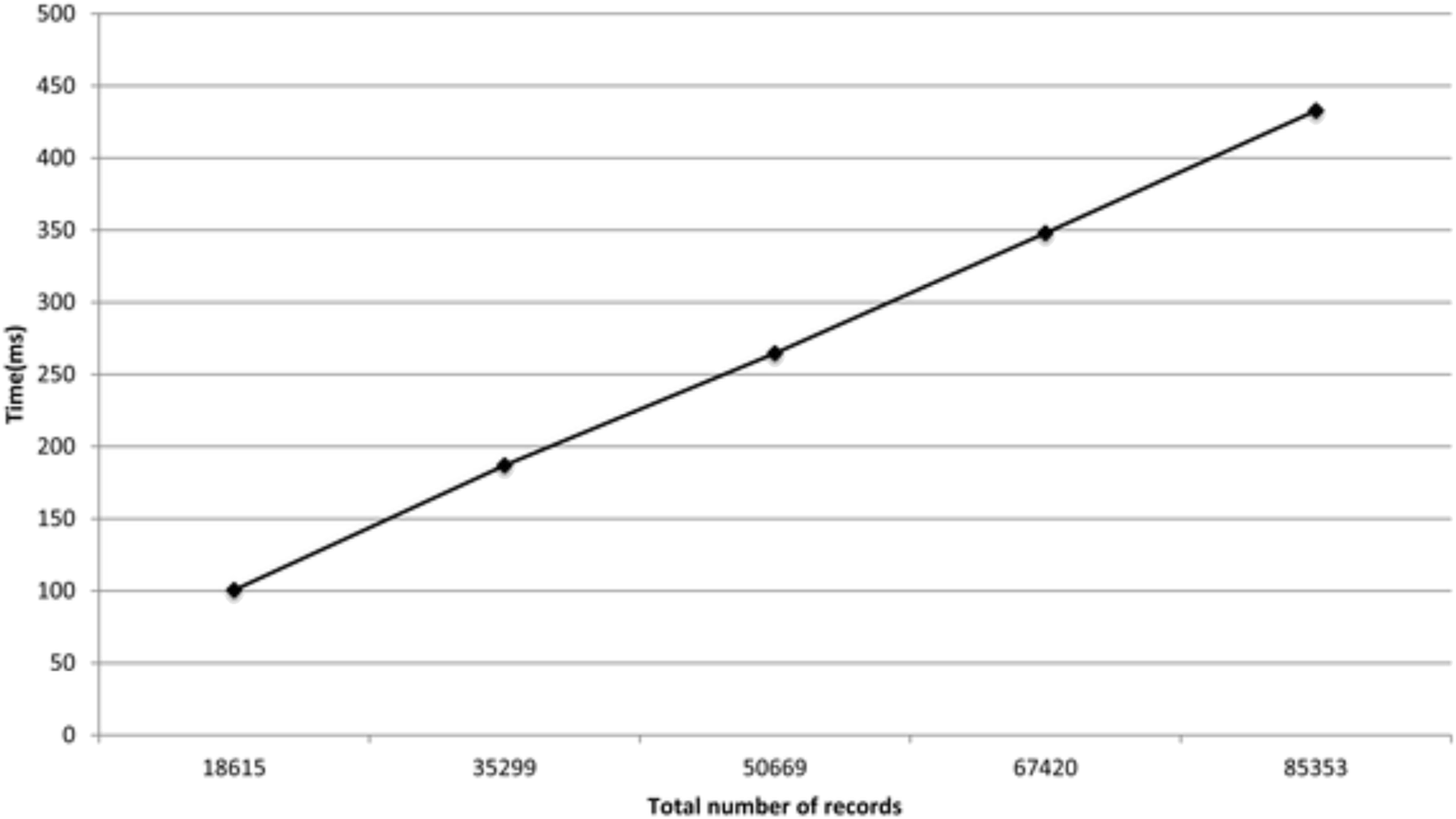


Fig. 10 The local computation time for the coordinator as the total number of records increases

Figures 11, 12, and 13 show the change in local computation time of Fürst, UNN, and NLSH, respectively, to perform their local computations as the total number of records increases. Note that Fürst was selected as the leader data custodian.

The number of records at Fürst, UNN, and NLSH were not equal. As a result, their local computation times were slightly different. In addition, some of the differences could be caused by the heterogeneity of their local computation resources.


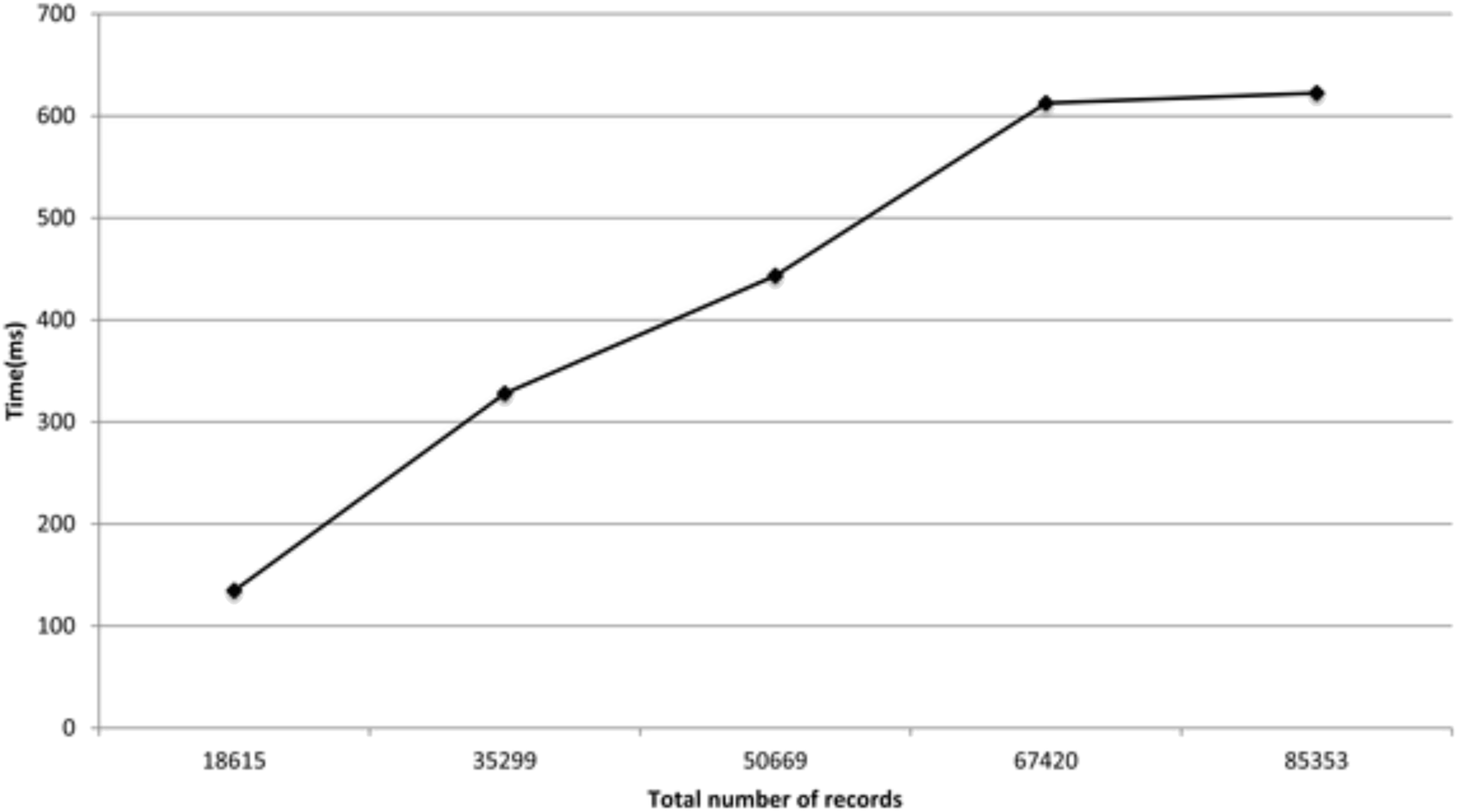


Fig. 11 The local computation time for Fürst as the total number of records increases


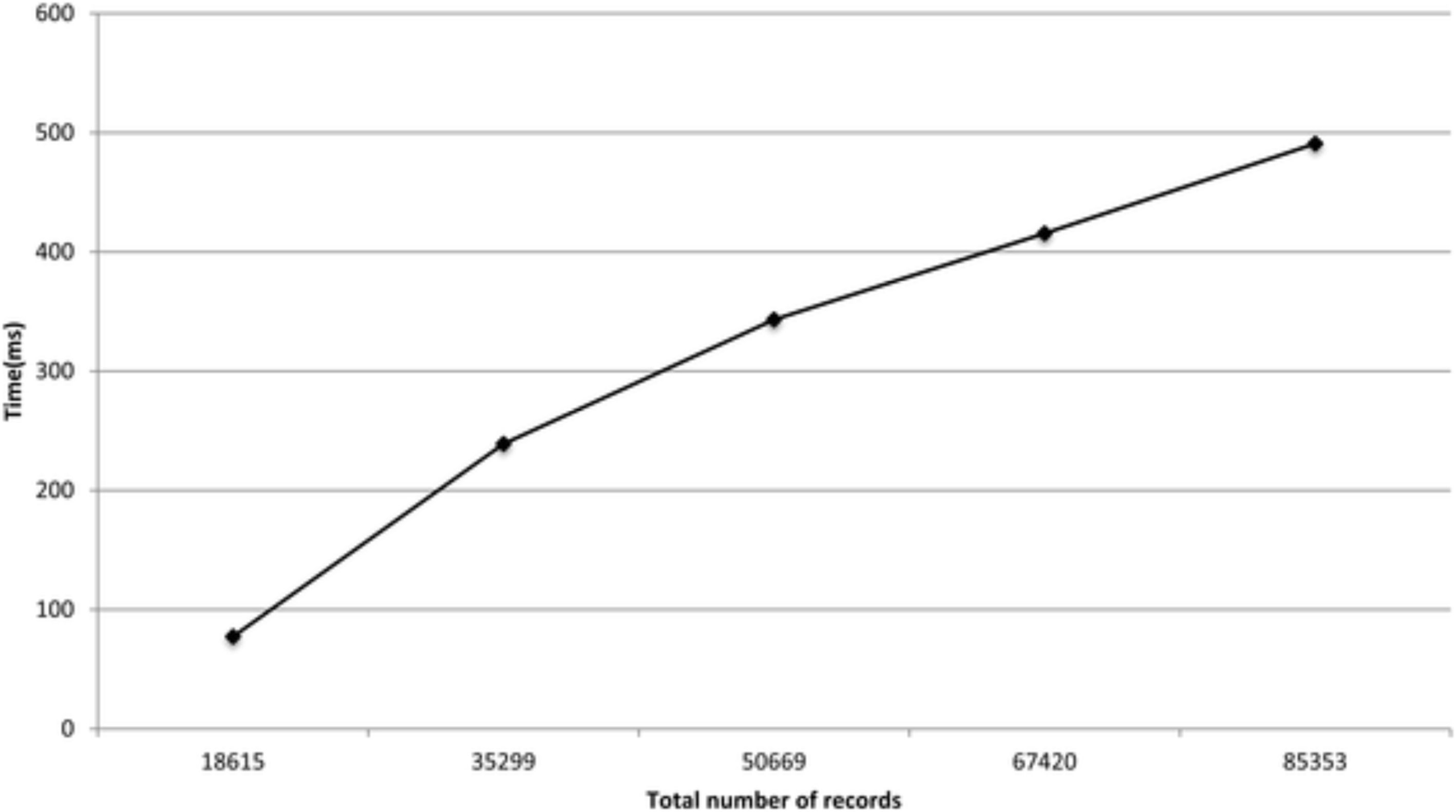


Fig. 12 The local computation time for UNN as the total number of records increases


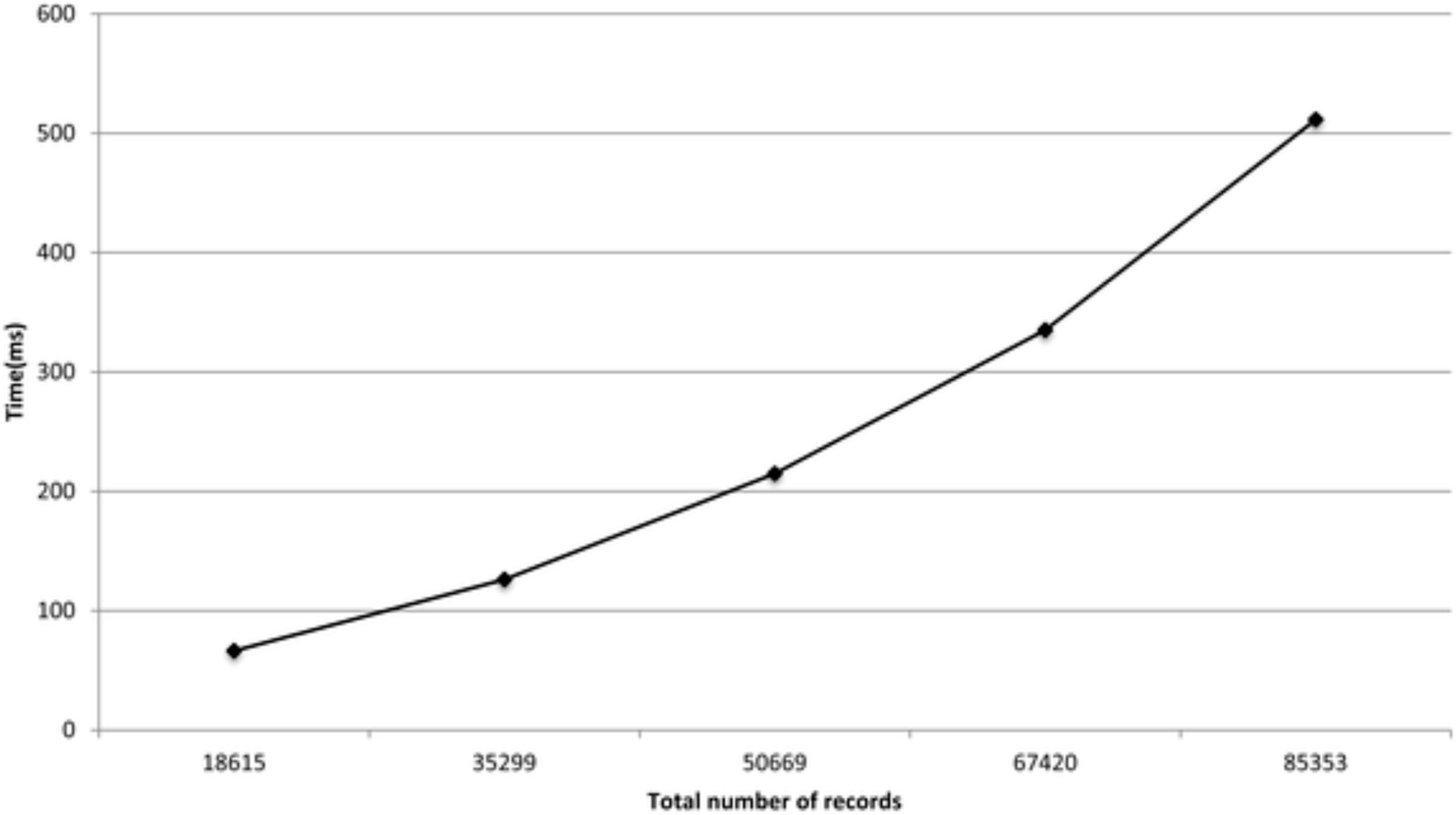


Fig. 13 The local computation time for NLSH as the total number of records increases

## In vitro experiments

In this section, we present the local computation times of the coordinator and the data custodians for the experiment ran on the simulated virtual datasets. Figure 14 shows the change in computation time of the *coordinator* to perform its local computations as the total number of records and number of data custodians increases.


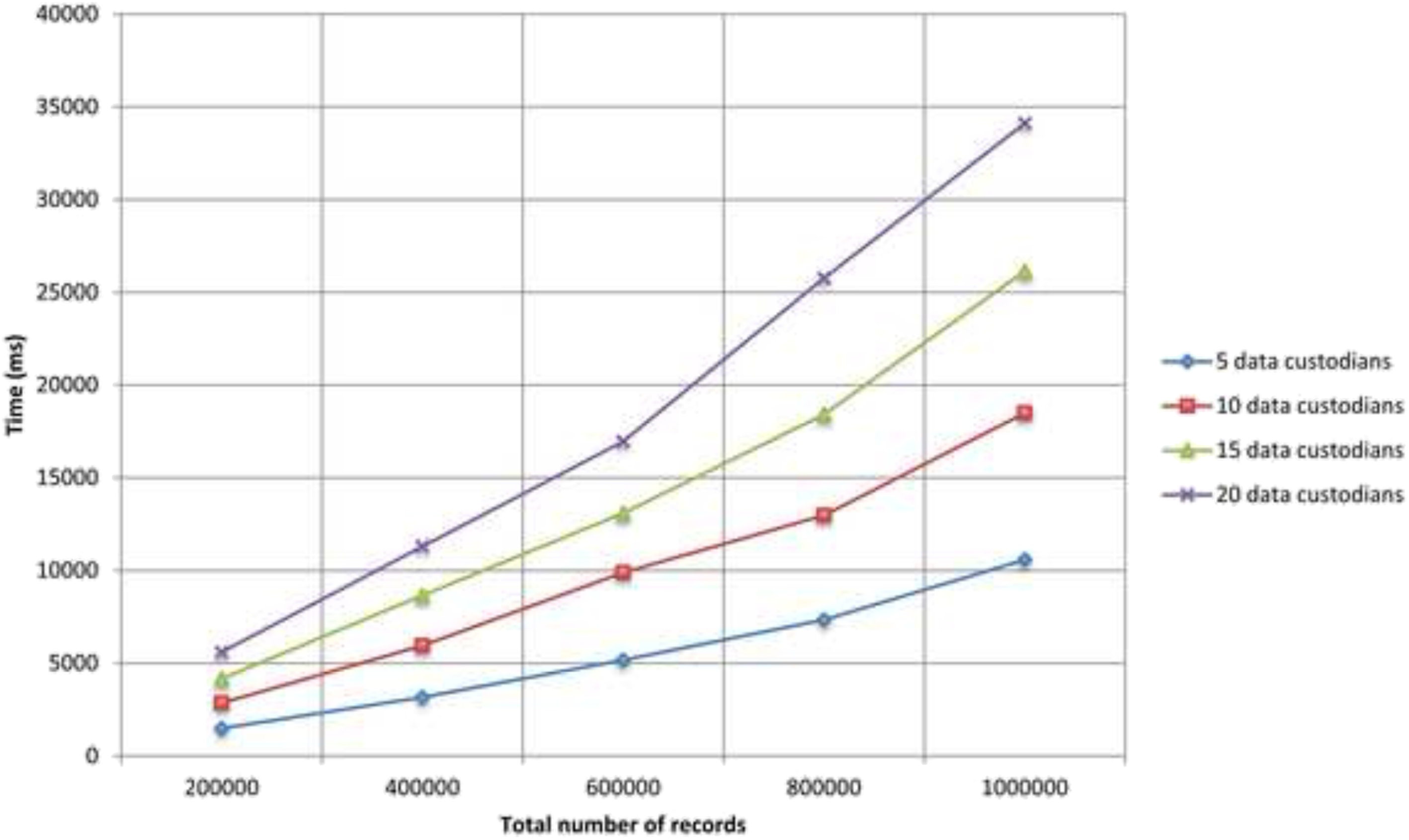


Fig. 14 The local computation time for the coordinator on the simulated datasets as the total number of records increases

Figure 15 shows the local computation time of the *coordinator* as the number of participating data custodians increases. Figures 16 and 17 show the local computation times of a leader data custodian and an ordinary data custodian, respectively, as the total number of records and participating data custodians increases.


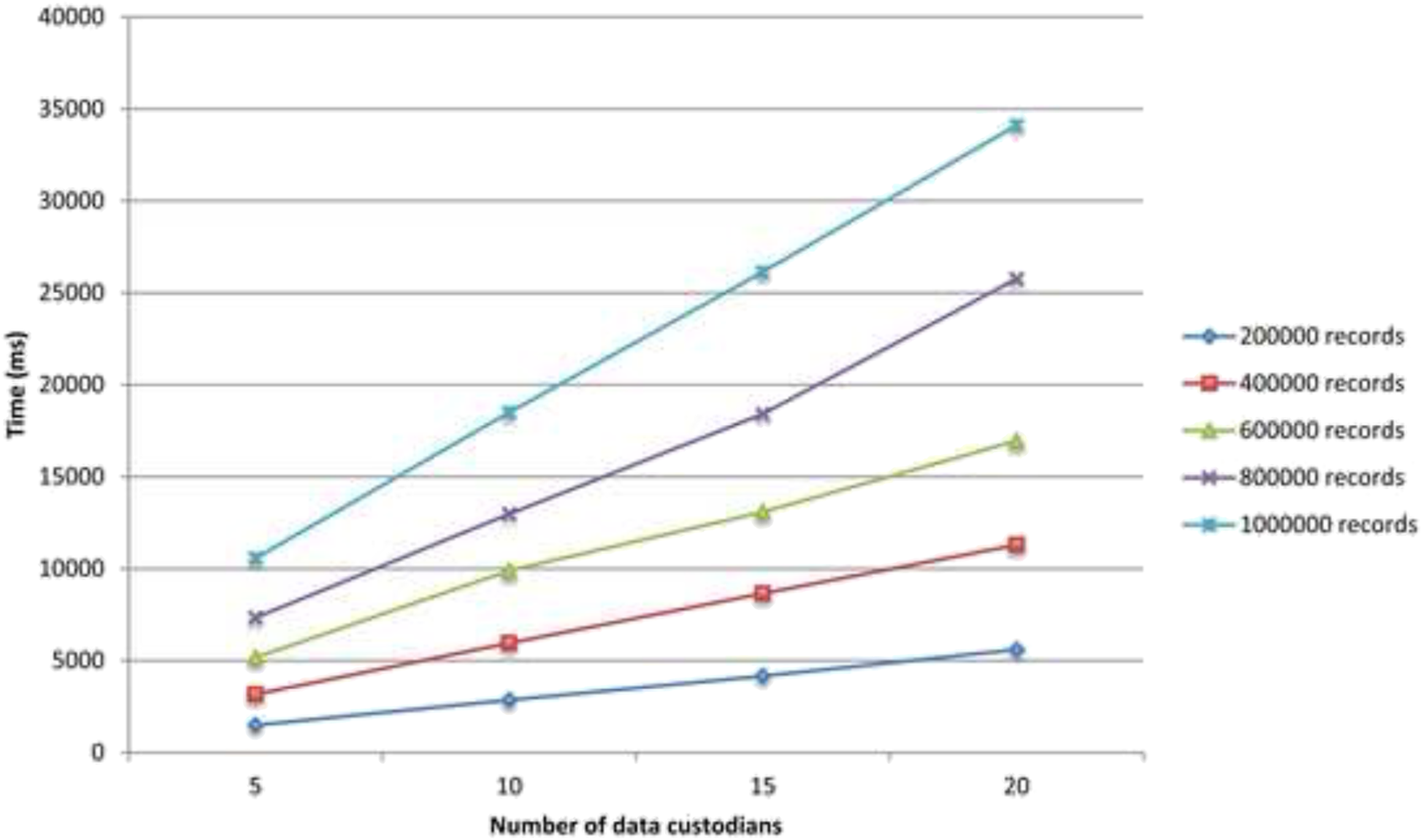


Fig. 15 The local computation time for the coordinator on the simulated datasets as the number of participating data custodians increases


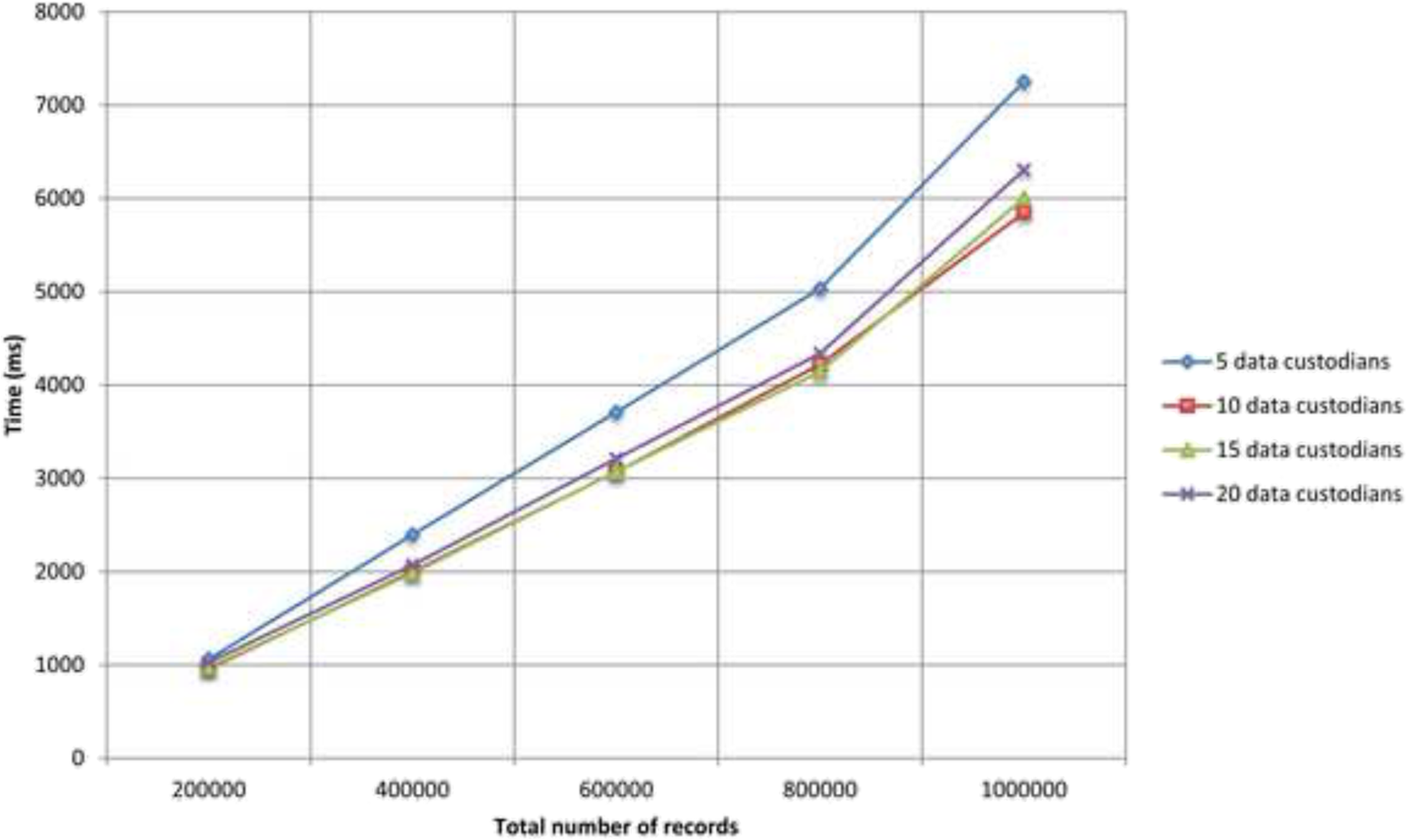


Fig. 16 The local computation time for the leader data custodian on the simulated datasets as the total number of records increases


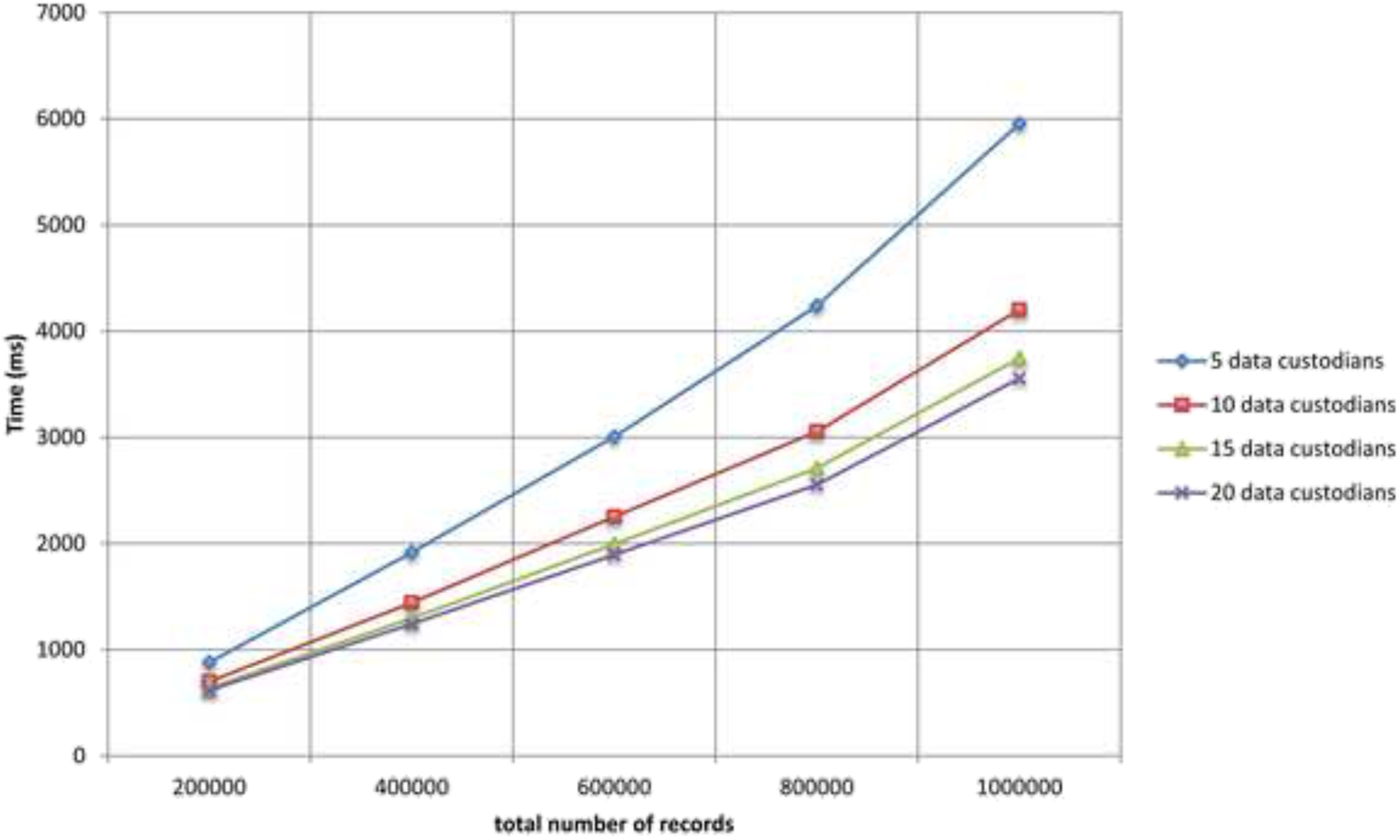


Fig. 17 The local computation time for a data custodian on the simulated datasets as the total number of records increases

Since the total number of records was equally distributed between all the participating data custodians, the local number of records of a data custodian increases when the number of participating data custodians decreases. Therefore, for the same total number of records, the local computation time of the leader data custodian (see Fig. 16) and an ordinary data custodian (see Fig. 17) slightly increases as the number of data custodians decreases.

We measured the scalability of the leader data custodian and ordinary data custodians as the total number of records and data custodians increases. However, unlike the previous experiments the local number of records of each data custodian was 50 000 records. Therefore, for 5, 10, 15, and 20 data custodians, the total numbers of records are 250 000, 500 000, 750 000, and 1 000 000, respectively. Figure 18 shows the local computation times of a leader data custodian and an ordinary data custodian as total number of records and participating data custodians increases.


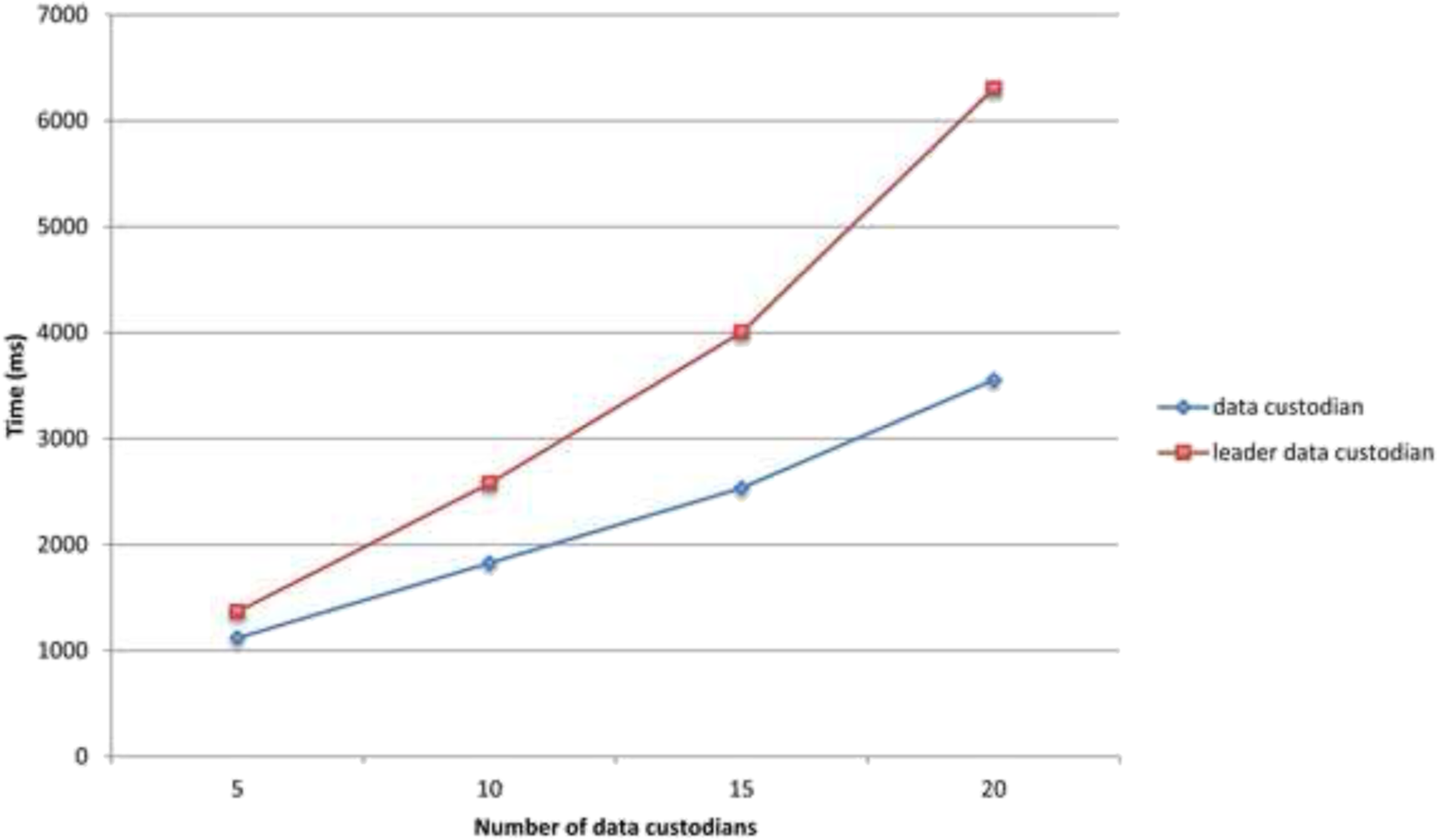


Fig. 18 The local computation time for the leader data custodian and a data custodian on the simulated datasets as the number of participating data custodians increases

# References

[1] Cohen S, Matias Y. Spectral Bloom Filters. Proc. 2003 ACM SIGMOD Int. Conf. Manag. Data, New York, NY, USA: ACM; 2003, p. 241–252. doi:10.1145/872757.872787.

[2] Kirsch A, Mitzenmacher M. Less hashing, same performance: Building a better Bloom filter. Random Struct Algorithms 2008;33:187–218. doi:10.1002/rsa.20208.

[3] El Emam K, HU J, Mercer J, Peyton L, Kantarcioglu M, Malin B, et al. A secure protocol for protecting the identity of providers when disclosing data for disease surveillance. J Am Med Inform Assoc 2011;18:212–7.

[4] Andersen A, Yigzaw KY, Karlsen R. Privacy preserving health data processing. 2014 IEEE 16th Int. Conf. E-Health Netw. Appl. Serv. Heal., IEEE; 2014, p. 225–30.

[5] Tarkoma S, Rothenberg CE, Lagerspetz E. Theory and practice of bloom filters for distributed systems. Commun Surv Tutor IEEE 2012;14:131–155.

1. There is complete separation between UNN and NLSH other than being on the same server. [↑](#footnote-ref-1)
